# Examining malaria treatment and prevention spending efficiency in malaria-endemic countries, 2000–2020

**DOI:** 10.1186/s12936-024-05165-w

**Published:** 2024-11-09

**Authors:** Angela E. Apeagyei, Ian Cogswell, Nishali K. Patel, Kevin O’Rourke, Golsum Tsakalos, Joseph L. Dieleman

**Affiliations:** 1 Institute for Health Metrics and Evaluation, Hans Rosling Center, 3980 15th Avenue NE, Seattle, WA 98195 USA; 2 Department of Health Metrics Sciences, School of Medicine, University of Washington, Seattle, WA 98195 USA

**Keywords:** Malaria, Health financing, Government expenditure, Development assistance for health, Spending

## Abstract

**Background:**

In 2021, an estimated 750,000 people died from malaria. Despite this significant burden, globally, malaria incidence and mortality rates have substantially dropped over the last 30 years. However, growth in spending on malaria and improved outcomes have recently stagnated. This development has made it more important than ever to understand what constitutes efficient spending on malaria.

**Methods:**

Data from various sources, including disaggregated data on malaria spending from the WHO Global Malaria Programme, National Health Accounts, and the Global Burden of Disease 2021 study was used in this study. The National Health Account report is produced at the end of a national accounting exercise that aims to map the flow of financial resources from all perspectives—incl. sources, agencies—in the health sector. Malaria spending estimates for all malaria-endemic countries from 2000 to 2020, with government and donor spending disaggregated into 11 key programme areas were generated in this study. Then, these spending estimates were combined with outcome data and estimated country efficiency using robust non-parametric stochastic frontier analysis and linear regression to examine the types of malaria spending associated with better malaria outcomes.

**Results:**

Across malaria-endemic countries, there is wide variation in malaria spending, with spending associated with the malaria burden within the country. Argentina, Paraguay, and Turkmenistan stood out as examples of low spending relative to their respective malaria incident per person at risk rates, while the Philippines, Guatemala, and Sri Lanka stood out as countries with case fatality ratios that were low relative to their malaria spending. Having a greater proportion of malaria spending sourced from donors or on prevention was associated with increases in incidence efficiency, while having a greater proportion of spending on anti-malarial medicines was associated with increases in case fatality efficiency.

**Conclusions:**

Prioritization of spending on prevention, anti-malarial medicines, and health systems strengthening can fight incident cases and fatalities simultaneously, especially in resource-scarce, malaria-endemic countries. Furthermore, improving the availability, frequency of collection, and quality of detailed disaggregated spending data is essential to support work that strengthens the evidence base on spending efficiency and work that improves understanding of how spending on malaria could be leveraged to bridge gaps in equity across population groups.

**Supplementary Information:**

The online version contains supplementary material available at 10.1186/s12936-024-05165-w.

## Background

Malaria caused an estimated 750,000 deaths in 2021 [1]. Of those deaths, 700,000 were in sub-Saharan Africa, while nearly 60% of these malaria deaths occurred in children under 5. While the burden of malaria remains substantial, globally, malaria outcomes—incidence and mortality—have seen significant drops over the last 30 years. Between 1990 and 2021, the mortality rate in malaria-endemic countries dropped by 35%, from 18 to 12 deaths per 100,000 [1]. These achievements have been ascribed to the scale-up of better treatment and prevention protocols for malaria as well as a galvanizing of more resources dedicated toward malaria [2]. In addition to malaria’s prominent place within the set of Millennium Development Goals, major global health international agencies were established that have been at the forefront of the fight against malaria since the turn of the century—namely the Global Fund to fight AIDS, Tuberculosis and Malaria (Global Fund) and the President’s Malaria Initiative [3–5].

Nonetheless, despite the progress in the fight against malaria, there has been stagnation over the past decade both in progress reducing burden and in raising additional resources for malaria control and efforts to move toward elimination. Between 2011 and 2019, development assistance for malaria grew by 3.5% annually compared to 28.9% between 2000 and 2010. Similarly, government spending on malaria grew by 4.3% annually from 2000 through 2017. This has made it especially critical for countries to improve their understanding of what constitutes effective spending on malaria by understanding which types of spending on malaria result in improved outcomes. This is because there are many ways that funding for malaria can be spent—focusing on preventive strategies, enhanced treatment protocols, human resource development, investment in infrastructure, improvements to procurement management, or other broad contributions to health systems strengthening. It is, however, unclear which of these types of spending is most associated with reductions in malaria incidence and mortality.

There are currently few published papers focused on allocative efficiency for malaria, examining which types of spending lead to the most improved outcomes [6–8]. One of the studies used a detailed simulation exercise to assess various malaria prevention and treatment interventions and their associated outcomes in Nigeria, while the second used data envelope methods to examine malaria spending efficiency in sub-Saharan Africa. A third study used a budget optimization model, and country-specific data to illustrate the optimal combination of prevention strategies. The first study found that over 5 years, gains from allocative efficiency could avert about 84,000 deaths or 15.7 million malaria cases. These gains could be made by prioritizing funding to long-lasting insecticidal nets (LLINs), intermittent preventive treatment, behavioural communication programmes, and the expansion of seasonal malaria chemoprevention (SMC) in seasonal areas. The second study found that spending efficiency in malaria prevention and treatment outcomes could be improved, and the factors associated with spending efficiency gains were education, availability of human resources, temperature levels, and the proportion of children sleeping under bed nets. The third study found that in Ghana, introducing the malaria vaccine in addition to the preventive strategies (LLINs, SMC, IRS) will be the best way to reduce under-5 malaria mortality at the lowest cost. Neither of these studies disaggregated the specific type of spending—government or donor—that improved efficiency. This present study aims to add to this limited literature in two ways. First, the study develops, reports, and makes publicly available a comprehensive set of estimates spanning malaria-endemic countries that focuses on estimating spending on malaria, inclusive of government spending, donor spending, private insurance spending, and out-of-pocket spending. For the two sources of spending that are the largest—government and donor spending—we further disaggregate spending into 11 key programme categories, such as anti-malarial medicines, insecticide-treated bed nets (ITNs), and human resources and technical assistance. Second, the study combines these spending data with estimates of malaria outcomes—malaria incidence per person at risk and case fatality ratios—and examines the efficiency of malaria spending relative to malaria outcomes. The results of this study highlight the types of spending that align most with improved malaria outcomes as well as country exemplars that have low levels of inefficiency relative to their total spending on malaria.

## Methods

### Overview

Malaria spending estimates for all malaria-endemic countries spanning 2000 through 2020, with government and donor spending disaggregated into 11 key programme areas: anti-malarial medicines; communication and advocacy; diagnostics; human resources and technical assistance; infrastructure and equipment; insecticides and spraying materials; ITNs; monitoring and evaluation; planning, administration, and overheads; procurement and supply management; and other were generated in this study. The time frame covered in this study was influenced by data availability and key time periods in global health, the start of the Millennium Development Goals in 2000 and the start of the COVID-19 pandemic in 2020. Three steps were used to generate these estimates. The first step was estimating the total spending levels by financing source. This step draws heavily from previously published research, extending the estimates and drawing from additional input data [9]. The second step was estimating the fraction of government spending on malaria that is spent on each of the 11 programme areas. This step includes identifying, extracting, and harmonizing all available malaria spending estimates. Spatiotemporal Gaussian process regression was used to generate plausible time trends, borrow strength across countries, and estimate uncertainty, which is largest when there are no input data or when the input data are contradictory[10]. The third step was modifying the methods used at the Institute for Health Metrics and Evaluation (IHME) for tracking development assistance for malaria such that the programme areas available aligned with those used for this study. After comprehensive, disaggregated spending estimates for malaria were generated, these spending estimates were combined with epidemiological outcome data from the Global Burden of Disease 2021 study to estimate country-level efficiency, relative to peer countries, and then evaluated to determine which spending categories were more associated with high malaria program efficiency (frontier analysis).

Frontier analysis is an economic modelling method typically used to examine efficiency in the production process. The basic underlying idea behind this method is that it models the optimal output level for a product or service given an input level. This result from a frontier analysis is slightly different from what could be obtained from a typical ordinary least squares regression (OLS). This is because an OLS regression will produce the average output for a given level of input, while a frontier analysis produces the optimal output, not the average output. The frontier maps out the farthest boundary that touches all the highest input/output points (Fig. S1 in the supplementary appendix). Compared to data envelope analyses, which can also be used for efficiency analysis, frontier analysis is a preferred approach for this study because the estimates of marginal productivity from a data envelope analysis can be questionable in some instances.

In the context of the frontier analysis, the distance between a combination of input/output point and the frontier is defined as the measure of “inefficiency.” To use a firm-specific example, the gap between firm A’s input/output value and the frontier represents a measure of the additional output that could be produced if firm A’s production process was more efficiently organized, like firm B, whose production point is on the frontier and produces a higher output level with the same input level as firm A.

### Estimating total spending on malaria by source (part 1, step 1)

To estimate spending on malaria by financing source, we completed three steps. First, we estimated total government spending on malaria using data extracted from the World Health Organization (WHO) World Malaria Report, National Health Accounts (NHA) reports, the WHO Global Health Expenditure Database (GHED), and concept notes and proposals from the Global Fund. The national health accounts reports are produced at the end of a national accounting exercise that aims to map the flow of financial resources from all perspectives—incl. sources, agencies, functions—in an individual country’s health sector. 60.6% of data were available and 4.9% of observations were identified as outliers using Cook’s distance. Spatiotemporal Gaussian process regression was used to create a full time series of estimates. Second, out-of-pocket spending on malaria was estimated using 124 country-years of extracted data from NHA reports and three price–volume approaches [inpatient visit spending, outpatient visit spending, and anti-malarial medicines spending] to augment the input data with a focus on estimating spending for the treatment of malaria. Lastly, we estimated prepaid private spending (PPP) on malaria using 81 country-years of extracted data from NHA reports along with estimates of the other financing sources. We calculated the median proportion of PPP spending to non-PPP spending and applied that ratio to the spending estimates for each country-year. These approaches are explained in detail in previously published papers [9]. Total donor spending on malaria data were available through IHME’s Financing Global Health study.

To model estimates of government and out-of-pocket spending on malaria, we leveraged the potential covariates listed in Table 1. These variables were considered based on their previously established relationship with malaria spending in the literature.

**Table 1:** List and source of potential covariates considered for the analyses

Potential covariate	Source
Average latitude (absolute value)	IHME (Global Burden of Disease 2021 Study); 2000–2020
Average life expectancy at birth	IHME (Global Burden of Disease 2021 Study); 2000–2020
GDP per capita (log-transformed)	IHME (Financing Global Health database); 2000–2020
Healthcare Access and Quality Index	IHME (Global Burden of Disease 2021 Study); 2000–2020
Incidence rate of malaria	IHME (Global Burden of Disease 2021 Study); 2000–2020
Mean level of maternal educational achievement (years)	IHME (Global Burden of Disease 2021 Study); 2000–2020
Prevalence of malaria	IHME (Global Burden of Disease 2021 Study); 2000–2020
Proportion of population living in an urban area	IHME (Global Burden of Disease 2021 Study); 2000–2020
Proportion of population under the age of 5	IHME (Global Burden of Disease 2021 Study); 2000–2020
Socio-demographic Index	IHME (Global Burden of Disease 2021 Study); 2000–2020
Universal health coverage index	IHME (Global Burden of Disease 2021 Study); 2000–2020

### Disaggregating government spending on malaria (part 1, step 2)

To generate a complete series of disaggregated spending estimates, we obtained disaggregated malaria spending data from the Global Malaria Programme at the WHO. There were 12 spending categories of the disaggregated data. These were the 11 final programme areas and a category for spending on training. To produce estimates that aligned with the development assistance for malaria data, training was aggregated to the human resources and technical assistance programme area. Additionally, 45 country-years of data from NHA reports and 54 country-years of data from National Malaria Control Programme documents and reports were extracted. We note that data were not available for each year for all countries, hence the limited total country-years of data reported. 15.2% of data were available. Table S1 in the Appendix shows the time periods and availability of data used from the various data sources.

To ensure internal validity of the data, several checks were completed. These checks included manually reviewing the data to remove obvious outliers and erroneous values. These outliers were disaggregated values greater than the total spending value or values greater than the estimates of total health spending reported in the Financing Global Health report [11, 12]. The disaggregated data received were inclusive of development assistance spending, the development assistance for health database compiled by IHME was used to exclude a portion of values based on proportions of development assistance received by spending type and Global Burden of Disease super-region and rescaled the remaining data to the available WHO total government spending values provided in the dataset. The categories available in the disaggregated data were also mapped to any additional data that was obtained from the NHA sub-accounts. Table S2 in the Appendix includes the categories that were aligned.

A spatiotemporal Gaussian process regression was used to model, fill in missingness, and generate a complete time series that covered the period of the study [13]. Spatiotemporal Gaussian process regression is a modelling approach that leverages available data, data from neighbouring countries and years, and specified covariates to generate estimates of spending. For covariate selection, a linear mixed effects model was used to estimate all combinations of covariates and selected only the models with lowest Akaike information criterion and Bayesian information criterion values. A tenfold cross-validation with out-of-sample predictions on these selected models was then completed. Root mean square error was used to select the best models. Cook’s distance was used to remove any remaining outlier observations (3.4% of data). The final covariates used in modelling each of the disaggregated components is provided in Table S6 in the Appendix.

### Disaggregating donor support for malaria (part 1, step 3)

Development assistance for malaria—in-kind and financial resources transferred from primary development channels to low- and middle-income countries—data were extracted from IHME’s Development Assistance for Health database [14]. The development assistance data covered the same time period, and was aligned with the disaggregated program areas in the government spending data to facilitate comparison. Specifically, malaria treatment in the database was aggregated with antimicrobial resistance to align with anti-malarial medicines in government spending. Health systems strengthening not classified as human resources or monitoring and evaluation in the database was equally redistributed to align with infrastructure and equipment; planning, administration, and overheads; and procurement and supply management in government spending. Spending data were adjusted for inflation and subsequently exchanged to 2021 US dollars. Conversions were performed using inflation and exchange rates based on those from the International Monetary Fund [15]. The methods for generating the IHME DAH data are described in detail elsewhere [11, 12]. Table S2 in the Appendix presents how the existing programme areas in the DAH dataset were mapped to the disaggregated government spending programme areas. The data used to perform the regression analyses excluded in-kind resources.

### Estimating efficiency in malaria spending and outcomes (part 2)

The analyses conducted for this study were completed in three steps. First, a frontier using robust linear meta-regression techniques developed at IHME was used to estimate the changes in 10-year malaria outcomes relative to malaria spending [16]. This linear meta-regression technique was preferred to other existing frontier methods because this technique did not require prior knowledge of the precise shape of the frontier, although priors that more spending was associated with better outcomes were set for this study. The outcome variables used in the study were malaria incidence per person at risk and case fatality. Case fatality was defined as deaths per incident case. Countries with zero incident cases or deaths in 2000, 2010, or 2020 and incidence and case fatality rates below the 5th percentile were excluded from the study. These exclusions were done to ensure that the study dataset focused on countries with comparable malaria burden. The frontier analyses included 185 and 181 data points for incidence and case fatality, respectively. However, seven (incidence) and nine (case fatality) data points were trimmed through fitting the linear meta-regression. In all modelling approaches, outliers present a problem. The trimming process is how the problem presented by outliers is addressed. This is important because outliers can disproportionately influence the location of the frontier.

Second, utilizing the estimated frontier, country-specific inefficiency values were calculated. These inefficiency values captured a country’s distance (in terms of change in incidence or case fatality) from the optimal values, relative to a country’s level of malaria spending. The further a country was from the frontier, the greater the estimated inefficiency (Supplementary Appendix p. 22).

The third step was to perform linear regressions to identify which spending programme areas—prevention, an aggregate of ITNs and insecticide and spraying materials (only the incidence regression); anti-malarial medicines (only the case fatality regression); diagnostics; and an aggregate of the remaining categories (except human resources and other)—and financing sources, government and development assistance, are associated with greater efficiency. These analyses were conducted to understand the specific types of spending programme areas and funding sources that spurred more efficient malaria outcomes.

Other data sources used to complete this analysis included national estimates of population at risk of malaria infection and preliminary national malaria incident cases and deaths data. The former were extracted from the WHO World Malaria Report (2000–2020), and the latter data were extracted from the Global Burden of Disease 2021 study (2000–2020) [17]. These malaria incident cases and death data were preliminary estimates from the Global Burden of Disease 2021 study. The methodology between GBD 2019 and 2021 was the same except for the inclusion of a COVID-19 adjustment for the incidence results. Estimates of deaths attributed to malaria with an adjustment for the COVID-19 pandemic were not available at the time of the analysis, so we leveraged unadjusted estimates. Briefly, the COVID-19 adjustment was derived from the PULSE surveys conducted by WHO and were applied to anti-malarial effective treatment rates, which are used in the incidence estimation process. This adjustment was applied to 33 African countries for 2020. See Annex 1 of the 2022 World Malaria Report and Dzianach et al*.* for further details on how the adjustments were derived [18–22].

## Results

**Fig. 1:**
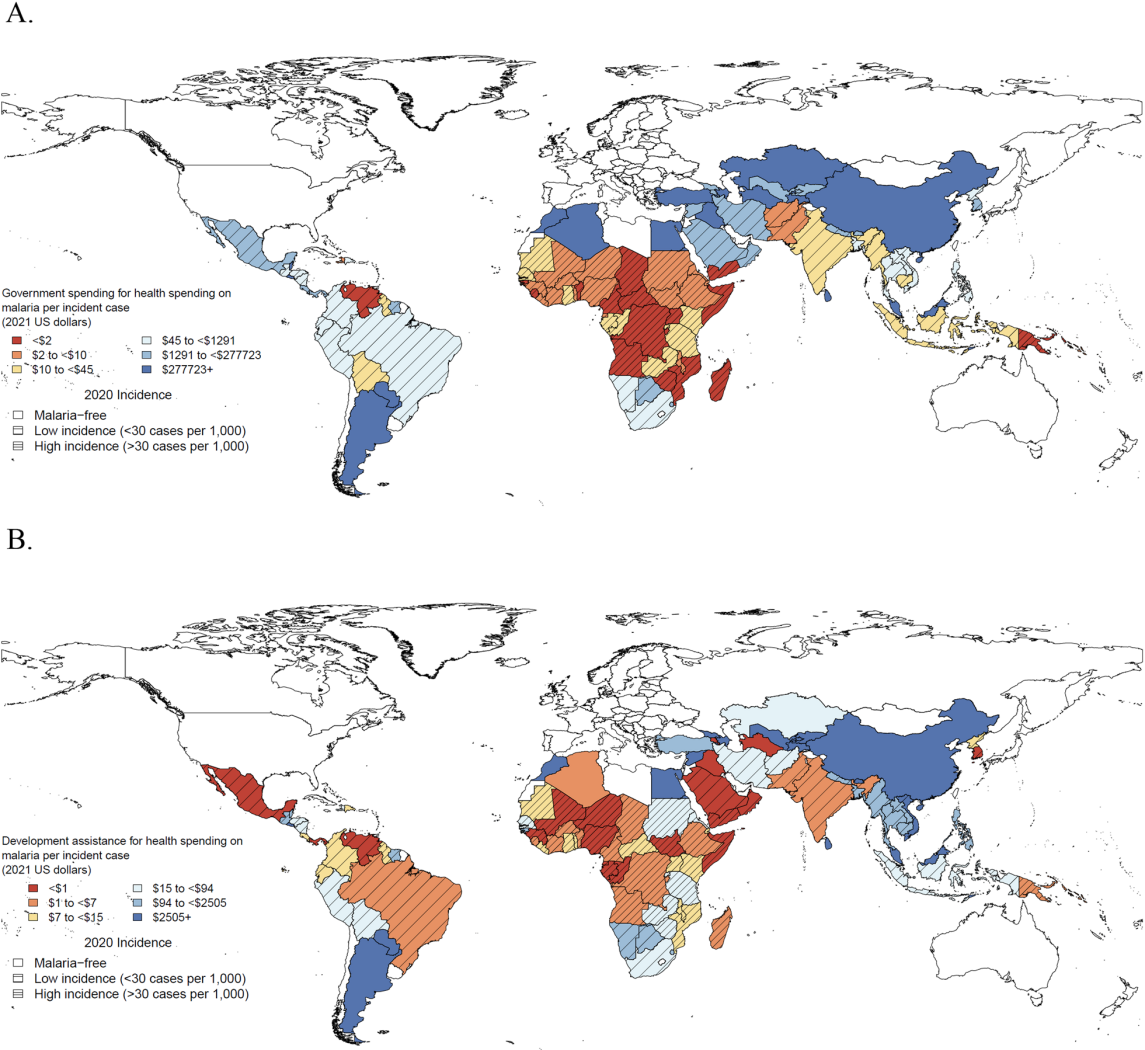
**A** Government spending on malaria per incident case by malaria endemicity, 2020. **B** Development assistance for malaria per incident case by malaria endemicity, 2020. Bins for spending on malaria per incident case are sextiles. All spending estimates are reported in 2021 USD per incident case. Countries with no incident cases were assigned an incidence of 1 case for calculations. Thick lines indicate high incidence of malaria in 2020. Thin lines indicate low incidence of malaria in 2020. No lines indicate malaria-free in 2020

Across malaria-endemic countries, the amount of malaria spending (per incident case) was associated with the malaria endemicity within the country. Among countries with non-zero incidence, Fig. 1 (panel A) shows governments in countries with low incidence spent the most (median $404; range $0–$488,000) per incident case, while governments in countries with high incidence spent the least (median $4; range $0–$34). This pattern in spending aligns with what may be necessary due to the number of cases and the efforts required at the various stages of elimination across countries, with some eliminating countries spending a great deal because they have low incidence while some countries spend less because they have effectively eliminated malaria. This pattern in spending and endemicity is replicated in the patterns in the development assistance provided to malaria-endemic countries as well (panel B)—countries with the fewest cases receive the most DAH per case, but in many cases, this is very little DAH total.

**Fig. 2:**
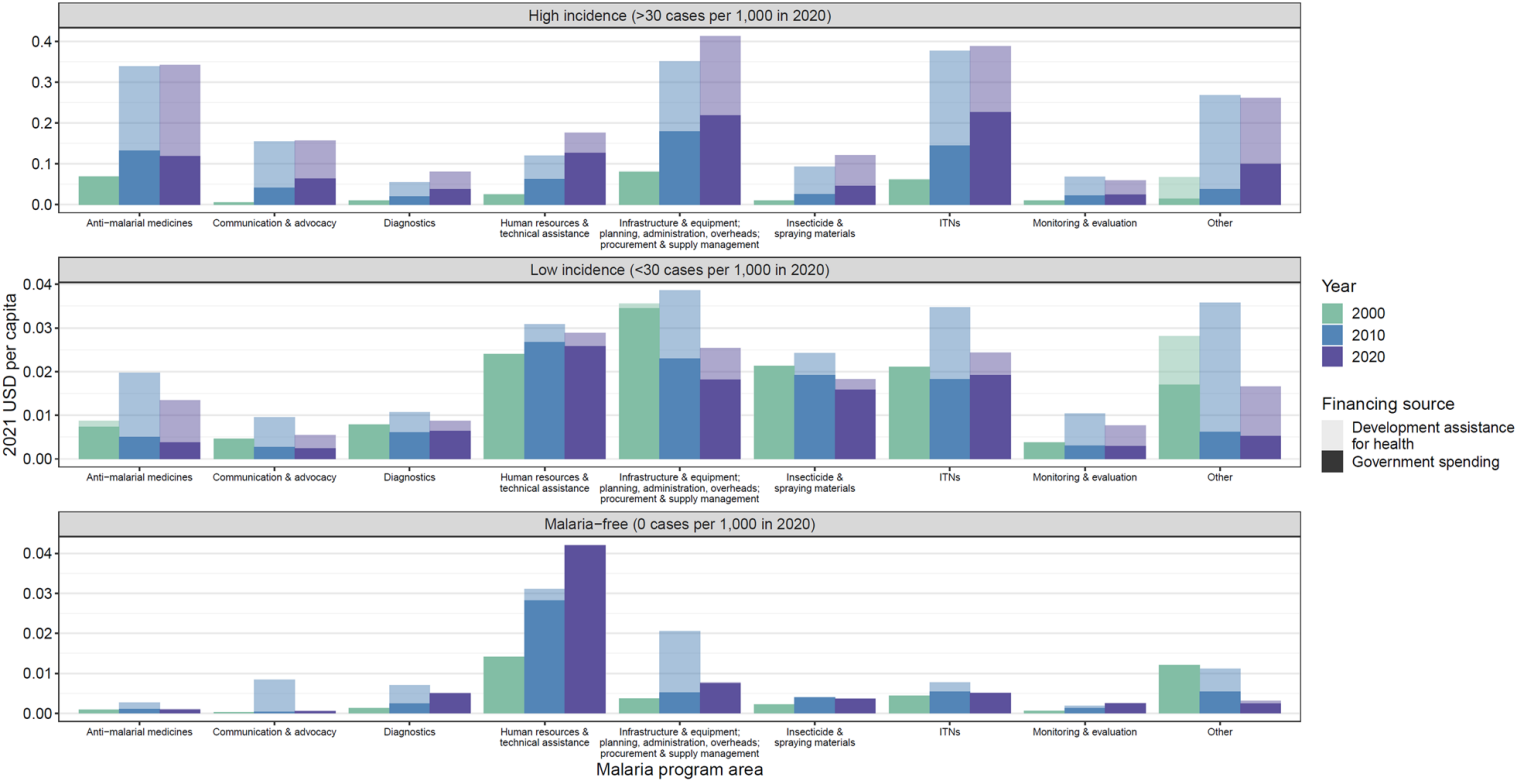
Malaria spending by endemicity, program area, and financing source; 2000, 2010, and 2020. All spending estimates are reported in 2021 USD per capita. Darker shades indicate government spending on malaria. Lighter shades indicate development assistance for malaria. *ITNs* insecticide-treated bednets

Figure 2 illustrates the association between the makeup of malaria spending over the past 21 years with endemicity. In 2020, according to spending from governments and DAH, countries with high incidence of malaria prioritized anti-malarial medicines (15.6%), ITNs (20.8%), and programme areas associated with health systems strengthening (21.4%). Countries with low incidence of malaria and malaria-free countries allocated the greatest proportion of spending to human resources and technical assistance at 22.0% and 59.9%, respectively. Spending on ITNs in countries with high incidence of malaria grew 8.4% annually over the study period. However, spending on ITNs in countries with low incidence of malaria and malaria-free countries grew less than 1% annually.

The following frontier lines were determined:

*Cℎange* in ln⁡(〖*incidence*〗_*t*) =  − 0.59 ln⁡(〖*THE*〗_(*t* + 10)) − 11.08.

*Cℎange* in ln⁡(〖case fatality〗_*t*) =  − 0.51 ln⁡(〖*THE*〗_(*t* + 10)) − 3.31.

**Fig. 3:**
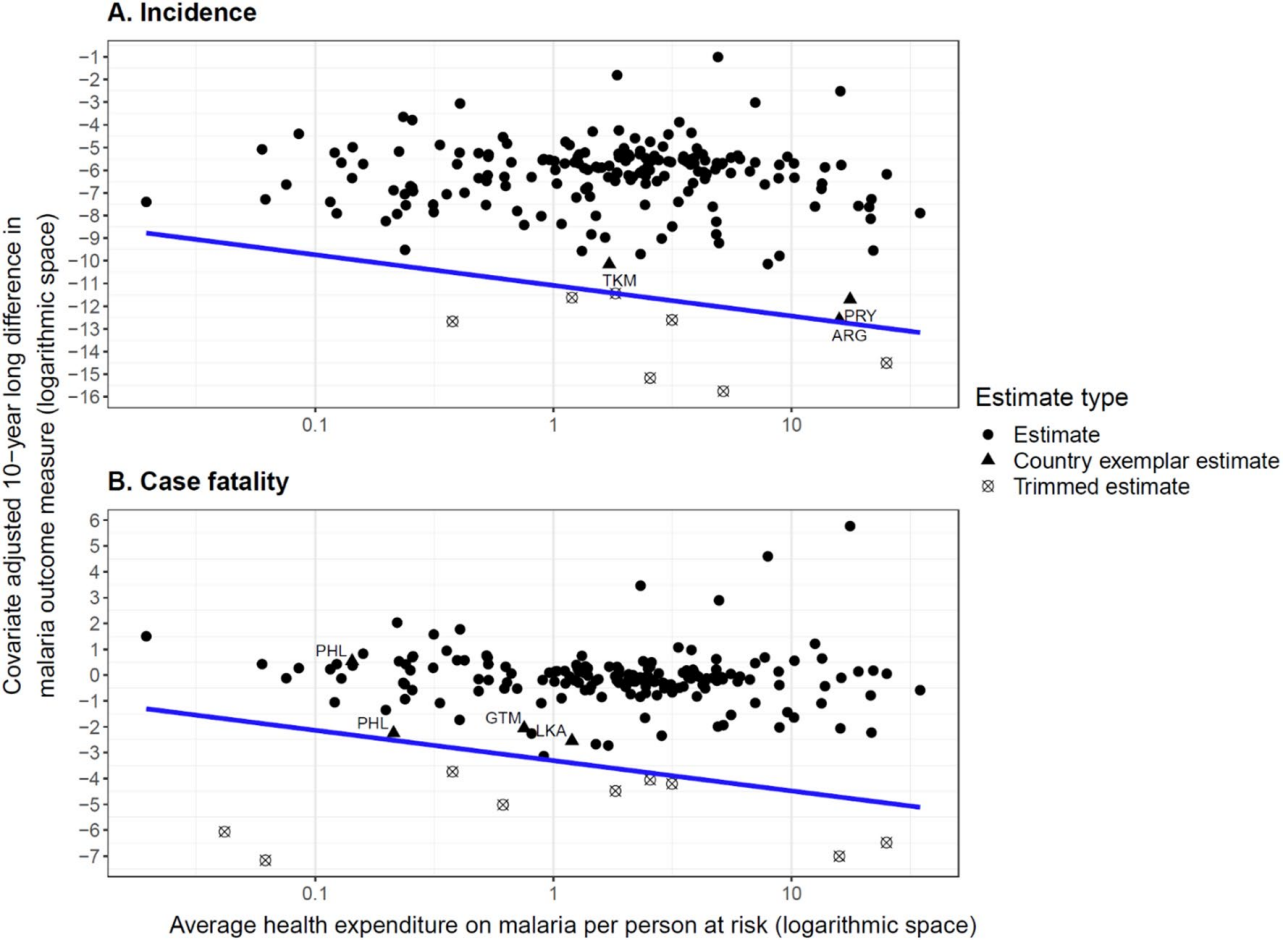
Graphic representation of frontier lines. Country exemplar estimates are indicated by triangles. Although, individual non-country-exemplar estimates may be closer to the frontier line than the country exemplar estimates, the overall inefficiency estimate is calculated as the mean of both inefficiency values for each 10 year period where available. *ARG* Argentina, *GTM* Guatemala, *LKA* Sri Lanka, *PHL* Philippines, *PRY* Paraguay, *TKM* Turkmenistan

These frontier lines are also presented graphically in Fig. 3a and b. The ranges of inefficiency values (vertical distance from the frontier) were 0.1 to 11.0 for incidence per person at risk and 0.1 to 10.5 for case fatality.

Table 2 lists three countries with the lowest mean inefficiency values as country exemplars for each outcome measure. For incidence, Argentina, Paraguay, and Turkmenistan were identified as the most efficient, and for case fatality, Sri Lanka, Guatemala, and the Philippines were identified as the most efficient. Timor-Leste and Djibouti had the greatest mean inefficiency values for incidence, and Azerbaijan and Paraguay had the greatest mean inefficiency values for case fatality.

**Table 2:** Top 3 countries with lowest mean inefficiency value

Rank	Country	Mean inefficiency value
Incidence		
1	Argentina	0.115
2	Paraguay	1.055
3	Turkmenistan	1.236
Case fatality		
1	Sri Lanka	0.825
2	Guatemala	1.083
3	Philippines	1.175

Table 3 reports the associations between inefficiency and the proportion of spending allocated to different financing sources (panel A) and programme areas (panel B). A greater proportion of spending on development assistance, prevention (aggregate of ITNs and insecticide and spraying materials) and health care access and quality were associated with increases in malaria incidence efficiency. In contrast, increases in malaria case fatality efficiency were associated with a greater proportion of spending on anti-malarial medicines and the later period (2011–2020) covered in this study. Neither prioritization of development assistance nor government funding as sources of malaria spending were associated with changes in case fatality efficiency.

**Table 3:** Results of linear regression analysis examining financing sources, programme areas, and malaria outcomes

	Dependent variable	
	Inefficiency in incidence	Inefficiency in case fatality
Panel A. Financing sources, malaria incidence, and case fatality		
Development assistance proportion	− 2.004^***^ (0.621)	− 0.862 (0.522)
Government spending proportion	− 1.050 (0.667)	0.597 (0.563)
HAQI	− 3.794^***^ (1.164)	− 1.153 (0.971)
2011–2020 period	0.941^***^ (0.224)	0.564^***^ (0.189)
Observations	167	165
Panel B. Programme areas, malaria incidence, and case fatality		
Anti-malarial medicines		− 2.599^**^ (1.138)
CAIEMEPAOPSM	− 1.601 (1.136)	− 0.946 (0.951)
Diagnostics	− 3.633 (3.408)	6.110^**^ (2.869)
Prevention	− 2.257^**^ (1.015)	
HAQI	− 3.921^***^ (0.890)	− 0.374 (0.797)
2011–2020 period	1.015^***^ (0.233)	− 0.703^***^ (0.191)
Observations	167	165

*CAIEMEPAOPSM* Communication and advocacy; infrastructure and equipment; monitoring and evaluation; planning, administration, and overheads; and procurement and supply management, *HAQI* Healthcare Access and Quality Index

*p < 0.1; **p < 0.05; ***p < 0.01

## Discussion

Spending on malaria has evolved over the past two decades. The establishment of the Global Fund and the focused attention generated by the Millennium Development Goals at the onset of the first decade all served to galvanize much-needed resources toward malaria [3, 4]. Subsequently, as countries have progressed through different stages of eradication over the study period, contrasting spending priorities have emerged. To better understand the malaria spending landscape, this study concentrated on producing estimates of type of spending and leveraging these estimates to determine their relationships with spending efficiency.

The programme area-specific spending estimates generated in this study highlight variations in malaria spending globally. Although countries with high incidence of malaria experience the greatest burden from the disease, their governments currently spend the least per incident case compared to countries with low incidence of malaria. This difference in spending may be further explained by high numbers of cases in countries with high burden of malaria and the income groups—predominantly low-income—in which these countries are classified. Conversely, for countries with low incidence of malaria, high spending may be necessary to diminish the likelihood of a resurgence and to fully eliminate malaria.

Furthermore, development assistance for malaria varies considerably across endemic countries as well. Countries with high incidence show on average greater funding received per incident case compared to countries with low incidence. This difference in spending is also likely because these countries face a higher burden in terms of incident cases. However, providing additional funds to countries with lesser burden could be necessary assistance to further elimination goals [23].

The changes observed in spending on malaria interventions over time highlight that countries prioritize their spending based on their stage of elimination. Countries with the greatest incidence prioritize anti-malarial medicines for treatment, ITNs for prevention, and health systems strengthening. In countries with little to no incidence, spending estimates show a greater prioritization of human resources. This is perhaps because of the many activities required to move a country toward elimination or malaria-free status. ITNs use grew immensely in high-incidence countries, while other countries showed little growth over the period of the analyses. This observed pattern in use of preventive interventions suggests that additional spending on prevention could further advance low-incidence countries into elimination.

Three countries—Argentina, Paraguay, and Turkmenistan—stood out as country exemplars whose new case rates relative to spending were less than expected in incidence efficiency, and the Philippines, Guatemala, and Sri Lanka were observed as exemplars in malaria case fatality efficiency. Arguably, various policies introduced or implemented within these countries have contributed to these outcomes. For efficiency in incidence, all three exemplar countries are countries that have been certified as malaria-free by the WHO. Argentina was declared malaria-free in 2009, Turkmenistan in 2010, and Paraguay in 2018. In Argentina, this achievement was realized following years of consistent investment in policies and interventions focused on fully integrating malaria prevention and treatment into the national health care system. Specifically, this included integrating malaria surveillance into the national surveillance system for febrile illness and integrating malaria treatment services into the primary health care systems, especially in areas with increased risk of resurgence [24]. Similarly, in Paraguay, first a five-year plan that prioritized community engagement on the prevention and treatment of malaria and active case management was developed and implemented. Subsequently, a three-year plan that focused on enhancing the skills of the frontline workers to prevent, detect, and treat malaria, especially severe cases, was instituted [25]. In Turkmenistan, elimination of malaria was achieved through active cross-border collaboration, sustained political commitment and funding, and the introduction of a national plan for malaria elimination [26]. Similarly, for efficiency in case fatality, the exemplar countries—the Philippines, Guatemala, and Sri Lanka—had all proactively embarked on increasing coverage for known effective malaria control interventions. For instance, the Philippines’ national malaria control programme had prioritized expanding coverage of insecticide-treated bed nets, LLINs, quality diagnostics, and effective artemisinin-based combination therapy [27]. Guatemala had also implemented a comprehensive package of services including vector control, surveillance, and case management to limit case fatality associated with malaria [28, 29]. Sri Lanka had eliminated malaria as of 2016 through a high-performing anti-malaria programme and consistent financial support from both government and donors [30].

The results from this study also highlight the efficiency gains from a comprehensive malaria control package. This is highlighted in the strategy used by all the exemplar countries found on the frontier in our study, as discussed in the preceding paragraph. These findings thus reiterate the importance of conducting more cost-effectiveness studies that assess the cost savings and efficiency gains from combining malaria control interventions in a comprehensive package as recommended for effective control [31]. Such studies will help make the investment case more clearly for countries that are exploring the cost-effective path to malaria elimination.

The regression results suggest associations between financing sources, types of malaria interventions, and efficiency in malaria incidence over the period covered in this study. Having a greater proportion of malaria spending sourced from development assistance and having a greater proportion of malaria spending focused on preventive interventions were associated with increases in incidence efficiency. This may be the result of specific spending categories such as ITNs and insecticide and spraying materials typically prioritized by external funders that emphasize prevention strategies [32, 33].

In contrast, the results on case fatality suggest associations between type of malaria spending and efficiency only. Financing source prioritization was not associated with efficiency in malaria case fatality. In other words, efficiency in malaria case fatality did not change whether a greater proportion of malaria spending was sourced from development partners or governments. However, prioritizing spending on anti-malarial medicines was associated with decreases in case fatality. These results have been corroborated in less expansive studies where findings suggest that expansion in treatment services and LLIN provision is key to reducing malaria mortality and incidence, respectively [6].

Furthermore, greater health care access and quality were related to increases in efficiency in incident cases and not in case fatality. This is an interesting result that suggests that countries with greater access to and quality of health care are efficient in reducing new cases of malaria over the study period. This finding is aligned with what is expected in health care systems that have the appropriate prevention strategies communicated to the populace and diagnostics capabilities readily available. Lastly, the 2011–2020 period was associated with decreased efficiency in incident cases, which could be the result of the plateauing in malaria spending witnessed by low- and middle-income countries (LMICs)—while for case fatality, the 10-year period was related to greater reductions, which aligns with increased access to anti-malarial medicines and better pharmaceuticals [34–36].

The estimates and analyses are subject to a few limitations. First, data sources for malaria spending disaggregated by source are sparse and difficult to align. As a result, most of the data for the disaggregation came from the WHO country-reported data described previously. While these are the best data available, it is important to acknowledge their limitations. To mitigate the impact of sparse disaggregated data, the WHO data was supplemented with data from other sources. These additional data sources were aligned as best according to the information provided. Nonetheless, there may be unknown discrepancies between sources which remain. Additionally, an acknowledgement that estimates of spending that are disease-specific are limited by the extent to which shared activities such as those carried out by the health personnel can be incorporated without double counting. Furthermore, the nature of malaria spending is complex, which makes identifying relationships between outcomes and spending complicated. For instance, high spending may be the result of a high burden, but it may also be the result of the availability of resources in wealthier countries and/or a concerted effort in a country to move from elimination to malaria-free without resurgence. As a result, comparing countries using malaria spending per person at risk can be difficult to fully interpret. Similarly, the efficiency in health systems may be largely influenced by the existing governance structures and financial management systems.

While a proxy measure of governance systems is included in the study as a robustness analysis, such aspects of a health system that matter for efficiency are difficult to quantify appropriately in studies. Lastly, the associations between type of spending and efficiency identified above are descriptive and not causal.

## Conclusion

Over the past two decades, malaria spending per person at risk has greatly increased—by 142% in malaria-endemic countries. However, much work remains to further reduce the burden of malaria in endemic countries as malaria remains the fourth-greatest cause of death globally in children under 5 and the second-greatest cause of death in sub-Saharan Africa. Additional prioritization of spending on prevention, anti-malarial medicines, and health systems strengthening has the potential to fight incident cases and fatalities simultaneously and efficiently in malaria-endemic countries. While the collection and dissemination of malaria financing data have improved in recent times, more frequently collected and detailed disaggregated spending data, if prioritized and available, would support work that strengthens the evidence base on spending efficiency and work that improves understanding of how malaria financing could be leveraged to bridge gaps in equity across population groups.

## Supplementary Information

Additional file 1.

## Data Availability

The datasets generated and analysed during the current study are not publicly available yet due to publication requirements. The dataset will be made publicly available online in the Global Health Data Exchange at https://ghdx.healthdata.org upon publication.

## Abbreviations

DAH: Development Assistance for Health
GBD: Global Burden of Disease
GHED: Global Health Expenditure Database
IHME: Institute for Health Metrics and Evaluation
IRS: Indoor Residual Spraying
ITN: Insecticide-treated bed nets
LLIN: Long-lasting insecticidal net
LMICs: Low- and middle-income countries
OLS: Ordinary least squares
PPP: Prepaid private spending
NHA: National Health Accounts
SMC: Seasonal malaria chemoprevention
WHO: World Health Organization
