# Supplementary material for: Examining malaria treatment and prevention spending efficiency in malaria-endemic countries, 2000–2020
Source: Malar J. 2024 Nov 9;23:333. doi: 10.1186/s12936-024-05165-w (PMC11550530; doi:10.1186/s12936-024-05165-w)
Supplement: Supplementary file 1 — Additional file 1. [file 12936_2024_5165_MOESM1_ESM.docx]

**Supplementary Methods Appendix**

**Examining malaria treatment and prevention spending efficiency in malaria-endemic countries, 2000-2020**

Version: July 5, 2024

# Table of contents

| **Section** | **Page** |
| --- | --- |
| S1: Overview  S2: Currency conversion | 3  4 |
| S3: Government health expenditure as a source on malaria | 5 |
| S3.1 Data  S3.2 Creating disaggregated government expenditure on malaria categories | 5  7 |
| S3.3 Modeling government expenditure on malaria patient care | 9 |
| S3.4 Modeling total government expenditure on malaria  S3.5 Modeling disaggregated government expenditure on malaria  S4: Spending efficiency analysis  S4.1 Data  S4.2 Frontier analysis  S4.3 Country exemplar analysis  S4.4 Inefficiency regression analysis  S5: Malaria incidence categorization of the 106 countries  S6: Data density  S7: GATHER Compliance  S8: References | 14  17  20  20  23  24  34  35  40  42  43 |
|  |  |
|  |  |
|  |  |

S1: Overview

This study is the continuation of previous research published in Haakenstad, A. et al., 2016. The methodology presented in that research and the accompanying supplementary appendix has been continued here with the addition of additional input data, extended spending estimates, disaggregated malaria spending estimates, and a spending efficiency analysis. Therefore, the methodology included in this supplementary appendix aligns with the methodology described in the previous study with the exception of the new updates.

S2: Currency conversion

All malaria expenditure estimates were produced and reported in 2021 United States dollars (USD). Data sources reported expenditure in either nominal local currency units (LCUs) or nominal USD. To convert nominal LCUs to USD, we applied deflators to nominal LCUs to inflate to 2021 LCUs. We then applied exchange rates to produce 2021 USD. When LCUs were not reported, we extracted reported expenditure in nominal USD, applied corresponding nominal exchange rates to produce nominal LCUs, inflated nominal LCUs to 2021 LCUs with deflators, and finally exchanged 2021 LCUs to 2021 USD. All deflators and exchange rates were extracted from the World Bank,^1^ International Monetary Fund,^2^ Penn World Tables,^3^ the United Nations National Accounts^4^ and the World Health Organization,^5^ and were imputed to provide a complete series for each of the variables between 1950 and 2021. We then used several models including ordinary least-squares regression and mixed effects models, to complete each source series. More information about the approach to converters and deflators may be found in Global Burden of Disease Health Financing Collaborator Network (2023).^6^

# S3: Government health expenditure as a source on malaria

## Data

Government spending on malaria was drawn from seven main sources. First, we extracted data on total government spending reported by National Malaria Control Programmes (NMCPs) to the World Health Organization (WHO) and published in the WHO’s annual World Malaria Reports (WMRs).^7^ Additionally, we conducted a web search of country Ministry of Health (MoH) websites for NMCP-related documents. We conducted a web search of all existing National Health Accounts, including those hosted by the WHO on the Global Health Expenditure Database, and found 80 reports that estimated total government expenditure as a source dedicated to malaria prevention, control, and treatment and 12 reports that estimated disaggregated government expenditure as a source.^8^ We extracted the government spending reported by governments submitting proposals and concept notes to the Global Fund. We excluded any projections for spending reported in these documents submitted to the Global Fund. We extracted domestic general government expenditure on malaria data from the Global Health Expenditure Database (GHED) on the WHO website. Additionally, we received country reported data on disaggregated government spending on malaria from WHO. Finally, we leveraged government budget for malaria data from the Pan American Health Organization (PAHO) Malaria Surveillance Indicators. Table 1 shows the number of countries-years available for each data source.

**Table S1: Main data sources for government health expenditure as a source for malaria**

| **Data source** | **Country-years of total government spending on malaria data** | **Country-years of disaggregated government spending on malaria data** |
| --- | --- | --- |
| NMCP reports in the WMRs | 1265 | 0 |
| NMCP documents from country MoHs | 0 | 16 |
| NHAs (System of Health Accounts) | 156 | 12 |
| Global Fund proposals and concept notes | 605 | 0 |
| Global Health Expenditure Database (GHED) | 269 | 0 |
| Country reported disaggregated government spending on malaria data to WHO | 0 | 779 |
| Pan American Health Organization (PAHO) Malaria Surveillance Indicators | 208 | 0 |

The spending reported by NMCPs to the WMRs typically capture expenditure on prevention activities, such as insecticide-treated nets, indoor residual spraying, and chemoprevention, as well as the purchase of antimalarial drugs and diagnostics. However, they do not include spending on patient care – e.g. spending on the labor, facilities, and other costs involved in providing care to malaria patients in government health facilities outside of drugs and diagnostics. Because the government spending numbers reported in the Global Fund concept notes and proposals are typically submitted by the same source – NMCPs – and aligned well with the reported numbers in the WMR, we assumed that this spending also did not include patient care.

## Creating disaggregated government expenditure on malaria categories

We leveraged our primary source of data (WHO) and the malaria-focused program areas from the development assistance for health (DAH) data from the Institute for Health Metrics and Evaluation in the creation of disaggregated government expenditure on malaria categories. Our primary data source informed what aspects of malaria expenditure we were reasonably able to quantify, and the malaria-focused DAH program areas informed necessary adjustments to allow comparability between the two sources of financing.

**Table S2: Aligning 11 final disaggregated government expenditure on malaria categories, 12 WHO disaggregated malaria expenditure categories, and 11 IHME malaria-focused DAH program areas**

| **Final disaggregated government expenditure on malaria categories** | **WHO disaggregated categories** | **IHME malaria-focused DAH program areas** |
| --- | --- | --- |
| Anti-malarial medicines | Anti-malarial medicines | Treatment + Drug resistance |
| Communication and advocacy | Communication and advocacy | Community outreach |
| Diagnostics | Diagnostics | Diagnosis |
| Human resources and technical assistance | Human resources and technical assistance + Training | HSS – human resources |
| Infrastructure and equipment | Infrastructure and equipment | (1/3) HSS – other |
| Insecticide and spraying materials | Insecticide and spraying materials | Indoor residual spraying |
| ITNs/PBOs | ITNs/PBOs | Nets |
| Monitoring and evaluation | Monitoring and evaluation | HSS – monitoring and evaluation |
| Planning, administration, overheads | Planning, administration, overheads | (1/3) HSS – other |
| Procurement and supply management | Procurement and supply management | (1/3) HSS – other |
| Other | Other | Other control + Other |

We prioritized the categorization from the WHO as disaggregating government expenditure was the primary focus of this study. We made one adjustment to the WHO data; we combined “Human resources and technical assistance” with “Training” to better align with the “HSS – human resources” program area, as splitting the program area was less feasible with the available information and both WHO categories were similar. We made multiple adjustments to the IHME malaria-focused program area data in aligning the two sources of financing. We aggregated “Treatment” and “Drug resistance” into “Anti-malarial medicines” as we assumed these program areas focused primarily on pharmaceutical interventions. “HSS – other” was split in thirds to align with “Infrastructure and equipment”, “Planning, administration, overheads”, and “Procurement and supply management”. We thought it important to retain these three categories in our final estimates as they are quite different. The keyword search implemented in the creation of malaria-focused DAH estimates for “HSS – other” aligned well with these three final categories. Finally, we aggregated “Other control” and “Other” to align with the “Other” WHO category. The control strategies included in the “Other control” DAH estimates did not align with any other more specific final category, and it was less feasible to disaggregate the “Other” WHO category with the available information. We continued our analysis with 11 final disaggregated government expenditure on malaria categories (Table S2).

## Modeling government spending on patient care

To estimate government spending on patient care, we built a price-volume model focused on spending on inpatient and outpatient care, respectively, as shown in (1).

𝐺𝑜𝑣𝑚𝑎𝑙 𝑝𝑎𝑡𝑖𝑒𝑛𝑡 𝑐𝑎𝑟𝑒 = (1)

(𝐺𝑜𝑣 𝑐𝑜𝑠𝑡 𝑝𝑒𝑟 𝑎𝑑𝑚𝑖𝑠𝑠𝑖𝑜𝑛𝑚𝑎𝑙 ∗ 𝑃𝑢𝑏𝑙𝑖𝑐 𝑎𝑑𝑚𝑖𝑠𝑠𝑖𝑜𝑛𝑠𝑚𝑎𝑙) +

(𝐺𝑜𝑣 𝑐𝑜𝑠𝑡 𝑝𝑒𝑟 𝑜𝑢𝑡𝑝𝑎𝑡𝑖𝑒𝑛𝑡 𝑣𝑖𝑠𝑖𝑡𝑚𝑎𝑙 ∗ 𝑃𝑢𝑏𝑙𝑖𝑐 𝑜𝑢𝑡𝑝𝑎𝑡𝑖𝑒𝑛𝑡 𝑣𝑖𝑠𝑖𝑡𝑠 𝑚𝑎𝑙)

In (2) - (5) below, we show the equations used to estimate each element of 𝐺𝑜𝑣𝑚𝑎𝑙 𝑝𝑎𝑡𝑖𝑒𝑛𝑡 𝑐𝑎𝑟𝑒. Estimates of 𝐺𝑜𝑣 𝑐𝑜𝑠𝑡 𝑝𝑒𝑟 𝑎𝑑𝑚𝑖𝑠𝑠𝑖𝑜𝑛𝑚𝑎𝑙, shown in (2), were based on country-specific average

𝑖𝑛𝑝𝑎𝑡𝑖𝑒𝑛𝑡 𝑢𝑛𝑖𝑡 𝑐𝑜𝑠𝑡 of all health conditions from Moses et al. (2018).^9^ We extracted estimates of malaria inpatient unit costs for 22 country-years from 13 peer-reviewed articles, as shown in Table 2.^10-22^ Only 11 country-years from these studies reported inpatient unit costs without spending on drugs and diagnostics. Therefore, we took the median share of non-drug, non-diagnostic spending and applied it to the remaining 11 country-years to generate 𝑚𝑎𝑙 𝑖𝑛𝑝𝑎𝑡𝑖𝑒𝑛𝑡 𝑢𝑛𝑖𝑡 𝑐𝑜𝑠𝑡 non drug & non diagnostic. We converted these values to 2021 US dollars and took the ratio of these values to the inpatient unit costs from Moses et al. (2018) in the country and year in which the study took place. The median of this ratio (.69) was applied to all inpatient unit costs to calculate 𝐺𝑜𝑣 𝑐𝑜𝑠𝑡 𝑝𝑒𝑟 𝑎𝑑𝑚𝑖𝑠𝑠𝑖𝑜𝑛𝑚𝑎𝑙.

### 𝐺𝑜𝑣 𝑐𝑜𝑠𝑡 𝑝𝑒𝑟 𝑎𝑑𝑚𝑖𝑠𝑠𝑖𝑜𝑛𝑚𝑎𝑙 = (2)

𝑖𝑛𝑝𝑎𝑡𝑖𝑒𝑛𝑡 𝑢𝑛𝑖𝑡 𝑐𝑜𝑠𝑡 ∗ 𝑚𝑒𝑑 (𝑚𝑎𝑙 𝑖𝑛𝑝𝑎𝑡𝑖𝑒𝑛𝑡 𝑢𝑛𝑖𝑡 𝑐𝑜𝑠𝑡 non drug & non diagnostic)

𝑖𝑛𝑝𝑎𝑡𝑖𝑒𝑛𝑡 𝑢𝑛𝑖𝑡 𝑐𝑜𝑠𝑡

**Table S3: Malaria inpatient unit costs extracted from peer-reviewed literature**

| **Country** | **Year** | **Inpatient unit costs**  **(2021 USD)** | **Non-drug, non- diagnostic inpatient unit costs**  **(2021 USD)** | **Study** |
| --- | --- | --- | --- | --- |
| Bangladesh | 2003-2005 | 60.9 |  | Lubell et al. 2009 |
| Cameroon | 2013-2014 | 69.09 | 39.08 | Maka et al. 2016 |
| China | 2014 | 926.17 | 175.11 | Tang et al. 2017 |
| Democratic Republic  of the Congo | 2005 | 37.97 |  | Tsakala et al. 2005 |
| Ghana | 2009 | 29.37 |  | Sicuri et al. 2013 |
| India | 1996-98 | 27.95 | 18.66 | Gogtay et al. 2003 |
| India | 2003-2005 | 67.32 |  | Lubell et al. 2009 |
| Indonesia | 2003-2005 | 72.25 |  | Lubell et al. 2009 |
| Kenya | 2004-2005 | 151.86 |  | Ayieko et al. 2009 |
| Kenya | 2015 | 86.51 |  | Rakuomi et al. 2017 |
| Kenya | 2009 | 29.17 |  | Sicuri et al. 2013 |
| Myanmar | 2003-2005 | 35.23 |  | Lubell et al. 2009 |
| Nigeria | 2009 | 96.44 | 87.04 | Lubell et al. 2011 |
| Papua New Guinea | 2007-2008 |  | 3.44 | Davis et al. 2011 |
| South Africa | 2001 | 408.75 |  | Muheki et al. 2004 |
| Tanzania | 2009 | 65.54 | 40.3 | Lubell et al. 2011 |
| Tanzania | 2009 | 20.12 |  | Sicuri et al. 2013 |
| Thailand | 2001 | 211.67 | 110.57 | Kyaw et al. 2014 |
| Uganda | 2009 | 56.54 | 46.88 | Lubell et al. 2011 |
| Zimbabwe | 2000 | 3120.64 | 2912.34 | Hongoro and McPake  2003 |

𝑃𝑢𝑏𝑙𝑖𝑐 𝑎𝑑𝑚𝑖𝑠𝑠𝑖𝑜𝑛𝑠𝑚𝑎𝑙, shown in (3), represents the number of inpatient admissions for malaria in government-run health facilities. NMCP programs reported the total number of 𝑖𝑛𝑝𝑎𝑡𝑖𝑒𝑛𝑡 𝑐𝑎𝑠𝑒𝑠𝑚𝑎𝑙 to the WHO (not disaggregated by the public and private sector but capturing admissions in both sectors) and they are published in WMRs annually. Some missingness characterizes these reported values – malaria inpatient cases were available for 35% of country-years in our sample of 106 countries. For this reason, we modeled logit-transformed malaria admissions as a share of total admissions (from Moses et al. 2018) with spatiotemporal Gaussian process regression (ST-GPR) and the following covariates: malaria Lysenko 5 from the Global Burden of Disease (GBD) study 2017,^23^ natural log transformed ten- year lag-distributed income per capita (LDI per capita), whether a country had a policy of providing artemisinin-based combination therapy (ACT) free-of-charge in the public sector as reported in WMRs, and random effects on country, region, and super-region. We back-transformed the dependent variable and multiplied the ratio with 𝑖𝑛𝑝𝑎𝑡𝑖𝑒𝑛𝑡 𝑐𝑎𝑠𝑒𝑠𝑚𝑎𝑙 from Moses et al. (2018) to produce total malaria inpatient admissions for each of the 106 countries in our sample over 2000-2020.

### 𝑃𝑢𝑏𝑙𝑖𝑐 𝑎𝑑𝑚𝑖𝑠𝑠𝑖𝑜𝑛𝑠𝑚𝑎𝑙 = (3)

𝑝𝑢𝑏𝑙𝑖𝑐 𝑡𝑟𝑒𝑎𝑡𝑒𝑑 𝑐𝑎𝑠𝑒𝑠𝑚𝑎𝑙

𝑖𝑛𝑝𝑎𝑡𝑖𝑒𝑛𝑡 𝑐𝑎𝑠𝑒𝑠_𝑚𝑎𝑙_ ∗ (

)

𝑡𝑟𝑒𝑎𝑡𝑒𝑑 𝑐𝑎𝑠𝑒𝑠𝑚𝑎𝑙

𝑝𝑢𝑏𝑙𝑖𝑐 𝑡𝑟𝑒𝑎𝑡𝑒𝑑 𝑐𝑎𝑠𝑒𝑠𝑚𝑎𝑙 were based on estimates of treatment-seeking in the public sector among children under five with a fever in the last two weeks, as estimated by Battle et al. (2016).^24^

𝑡𝑟𝑒𝑎𝑡𝑒𝑑 𝑐𝑎𝑠𝑒𝑠𝑚𝑎𝑙 were from the same source but focused on all treatment-seeking among children under five with a fever in the last two weeks. These are the only comprehensive set of estimates of malaria treatment-seeking available to-date. Furthermore, treatment-seeking rates for malaria among children have been shown to be similar to treatment-seeking among adults.^25,26^

To impute data for countries not included in the original study, we used a similar approach to Battle et al. (2016) – we modeled logit-transformed public treatment-seeking as a share of children under five with a fever in the last two weeks with ST-GPR and the following covariates: logit-transformed out of- pocket (OOP) expenditure as a share of total health expenditure from Global Burden of Disease Health Financing Collaborator Network (2019),^27^ log LDI per capita, coverage of the diphtheria-tetanus-pertussis vaccine, four antenatal care visits, and skilled birth attendance, and the sociodemographic index (SDI),

all from the GBD 2017 study, and random effects on country, region, and super-region.

𝑡𝑟𝑒𝑎𝑡𝑒𝑑 𝑐𝑎𝑠𝑒𝑠𝑚𝑎𝑙 were imputed with a linear mixed model with fixed effects on year and year squared and random effects on region and super-region. Since both 𝑝𝑢𝑏𝑙𝑖𝑐 𝑡𝑟𝑒𝑎𝑡𝑒𝑑 𝑐𝑎𝑠𝑒𝑠𝑚𝑎𝑙 and

𝑡𝑟𝑒𝑎𝑡𝑒𝑑 𝑐𝑎𝑠𝑒𝑠𝑚𝑎𝑙 were modeled as a share of fever cases, we multiplied these ratios by malaria incident cases from GBD 2021 to calculate counts of each measure. We assumed that the share of total admissions in the public sector is similar to the share of all treatment seeking that occurred in the public

sector and thus apply the ratio 𝑝𝑢𝑏𝑙𝑖𝑐 𝑡𝑟𝑒𝑎𝑡𝑒𝑑 𝑐𝑎𝑠𝑒𝑠𝑚𝑎𝑙 to 𝑖𝑛𝑝𝑎𝑡𝑖𝑒𝑛𝑡 𝑐𝑎𝑠𝑒𝑠

to estimate

𝑃𝑢𝑏𝑙𝑖𝑐 𝑎𝑑𝑚𝑖𝑠𝑠𝑖𝑜𝑛𝑠𝑚𝑎𝑙.

𝑡𝑟𝑒𝑎𝑡𝑒𝑑 𝑐𝑎𝑠𝑒𝑠𝑚𝑎𝑙

𝑚𝑎𝑙

Estimating 𝐺𝑜𝑣 𝑐𝑜𝑠𝑡 𝑝𝑒𝑟 𝑜𝑢𝑡𝑝𝑎𝑡𝑖𝑒𝑛𝑡 𝑣𝑖𝑠𝑖𝑡𝑚𝑎𝑙 , shown in (4), deploys a similar approach to

𝐺𝑜𝑣 𝑐𝑜𝑠𝑡 𝑝𝑒𝑟 𝑎𝑑𝑚𝑖𝑠𝑠𝑖𝑜𝑛𝑚𝑎𝑙 in (2). Estimates of 𝑜𝑢𝑡𝑝𝑎𝑡𝑖𝑒𝑛𝑡 𝑢𝑛𝑖𝑡 𝑐𝑜𝑠𝑡 for all health conditions were sourced from Moses et al. (2018).^28^ Malaria inpatient unit costs were extracted from 9 peer-reviewed articles.^29-37^ Only 5 country-years reported outpatient unit costs without spending on drugs and diagnostics. The median share of non-drug, non-diagnostic spending was thus applied to the remaining 4 country-years studies to generate 𝑚𝑎𝑙 𝑜𝑢𝑡𝑝𝑎𝑡𝑖𝑒𝑛𝑡 𝑢𝑛𝑖𝑡 𝑐𝑜𝑠𝑡 non drug & non diagnostic. We converted these values to 2018 US dollars and took the ratio of these values to the outpatient unit costs from Moses et al. (2018) in the country and year in which the study took place. The median of this ratio (.54) was applied to all outpatient unit costs to calculate 𝐺𝑜𝑣 𝑐𝑜𝑠𝑡 𝑝𝑒𝑟 𝑜𝑢𝑡𝑝𝑎𝑡𝑖𝑒𝑛𝑡 𝑣𝑖𝑠𝑖𝑡𝑚𝑎𝑙. Table 3 shows each of the point estimates extracted from peer reviewed literature.

### 𝐺𝑜𝑣 𝑐𝑜𝑠𝑡 𝑝𝑒𝑟 𝑜𝑢𝑡𝑝𝑎𝑡𝑖𝑒𝑛𝑡 𝑣𝑖𝑠𝑖𝑡𝑚𝑎𝑙 = (4)

𝑜𝑢𝑡𝑝𝑎𝑡𝑖𝑒𝑛𝑡 𝑢𝑛𝑖𝑡 𝑐𝑜𝑠𝑡 ∗ 𝑚𝑒𝑑 (𝑚𝑎𝑙 𝑜𝑢𝑡𝑝𝑎𝑡𝑖𝑒𝑛𝑡 𝑢𝑛𝑖𝑡 𝑐𝑜𝑠𝑡 non drug & non diagnostic)

𝑜𝑢𝑡𝑝𝑎𝑡𝑖𝑒𝑛𝑡 𝑢𝑛𝑖𝑡 𝑐𝑜𝑠𝑡

**Table S4: Malaria outpatient unit costs extracted from peer-reviewed literature**

| **Country** | **Year** | **Outpatient unit costs**  **(2021 USD)** | **Non-drug, non- diagnostic outpatient unit costs**  **(2021 USD)** | **Study** |
| --- | --- | --- | --- | --- |
| China | 2013-2014 | 1236.21 |  | Liu et al. 2016 |
| Ghana | 2009 | 3.08 |  | Sicuri et al. 2013 |
| Kenya | 2009 | 3.87 |  | Sicuri et al. 2013 |
| Nigeria | 2013 | 24.38 | 20.11 | Ezenduka et al. 2017 |
| Nigeria | 2016 | 15.57 | 1.60 | Salwu et al. 2016 |
| Papua New  Guinea | 2007-2008 |  | 0.517 | Davis et al. 2011 |
| South Africa | 2004 | 27.01 |  | Muheki et al. 2004 |
| Tanzania | 2003 | 2.55 |  | Njau et al. 2008 |
| Tanzania | 2009 | 1.89 |  | Sicuri et al. 2013 |
| Tanzania | 2005 | 6.64 | 5.51 | Wiseman et a. 2006 |
| Zambia | 2005 | 6.99 | 1.81 | Chanda et al. 2007 |

Estimates of 𝑃𝑢𝑏𝑙𝑖𝑐 𝑜𝑢𝑡𝑝𝑎𝑡𝑖𝑒𝑛𝑡 𝑣𝑖𝑠𝑖𝑡𝑠𝑚𝑎𝑙, shown in (5), was based on 𝑃𝑢𝑏𝑙𝑖𝑐 𝑎𝑑𝑚𝑖𝑠𝑠𝑖𝑜𝑛𝑠𝑚𝑎𝑙, estimated as in (3), and the 𝑝𝑢𝑏𝑙𝑖𝑐 𝑡𝑟𝑒𝑎𝑡𝑒𝑑 𝑐𝑎𝑠𝑒𝑠𝑚𝑎𝑙 which is an input to those estimates.

𝑃𝑢𝑏𝑙𝑖𝑐 𝑎𝑑𝑚𝑖𝑠𝑠𝑖𝑜𝑛𝑠𝑚𝑎𝑙 were subtracted from 𝑝𝑢𝑏𝑙𝑖𝑐 𝑡𝑟𝑒𝑎𝑡𝑒𝑑 𝑐𝑎𝑠𝑒𝑠𝑚𝑎𝑙 to calculate all outpatient visits for malaria in each country over 2000-2020.

### 𝑃𝑢𝑏𝑙𝑖𝑐 𝑜𝑢𝑡𝑝𝑎𝑡𝑖𝑒𝑛𝑡 𝑣𝑖𝑠𝑖𝑡𝑠𝑚𝑎𝑙= (5)

𝑝𝑢𝑏𝑙𝑖𝑐 𝑡𝑟𝑒𝑎𝑡𝑒𝑑 𝑐𝑎𝑠𝑒𝑠𝑚𝑎𝑙 − 𝑃𝑢𝑏𝑙𝑖𝑐 𝑎𝑑𝑚𝑖𝑠𝑠𝑖𝑜𝑛𝑠𝑚𝑎𝑙

## Modeling total government spending on malaria

Total government spending is the sum of 𝐺𝑜𝑣𝑚𝑎𝑙 𝑝𝑎𝑡𝑖𝑒𝑛𝑡 𝑐𝑎𝑟𝑒 as described above and the government spending from the NMCP programs, as reported in WMRs and Global Fund proposals and concept notes (𝑁𝑀𝐶𝑃 𝐺𝑜𝑣𝑚𝑎𝑙), appended by the government spending on malaria estimated in NHAs (𝑁𝐻𝐴 𝐺𝑜𝑣𝑚𝑎𝑙), as shown in (6).

𝐺𝑜𝑣 𝑡𝑜𝑡𝑎𝑙𝑚𝑎𝑙 = 𝑁𝑀𝐶𝑃 𝐺𝑜𝑣𝑚𝑎𝑙 + 𝐺𝑜𝑣𝑚𝑎𝑙 𝑝𝑎𝑡𝑖𝑒𝑛𝑡 𝑐𝑎𝑟𝑒 (6)

= 𝑁𝐻𝐴 𝐺𝑜𝑣𝑚𝑎𝑙

To estimate a full time series for all years and countries in our study, we modelled 𝐺𝑜𝑣 𝑡𝑜𝑡𝑎𝑙𝑚𝑎𝑙 as a share of total government spending on health with ST-GPR. We first considered the following covariates: malaria incidence, malaria prevalence, LDI per capita, coverage of indoor residual spraying (IRS), insecticide-treated nets (ITNs), and ACTs, the plasmodium falciparum parasite rate (PFPR), PFPR adjusted for IRS and ITNS, malaria Lysenko 1, 2 and 5 measures, one- and four-visit antenatal care coverage, the healthcare access and quality index (HAQI), the proportion of the population living in an urban area, coverage of skilled birth attendance (SBA), the sociodemographic index (SDI) and the universal health coverage (UHC) index. All the covariates measured as

a proportion were logit-transformed. We sourced all covariate estimates from the GBD Study 2021 and from Malaria Atlas Project.

Because the availability of covariate data is higher for sub-Saharan Africa and patterns of incidence and intervention strategies differ from other regions, we split the government malaria spending data

into two groups – sub-Saharan African and non-sub-Saharan African countries – and modeled each group separately. We performed covariate selection for each dataset, starting with the set

of 19 potential covariates.

We first conducted a lasso regression to determine which covariates were least correlated, conditional on other covariates, with the fraction of government spending on malaria as the dependent variable. Covariates with an estimated coefficient of zero were removed from the set of possible covariates. We then used linear mixed effects regression to estimate all models including all possible combinations of the remaining covariates.

We then selected the intersection of 1000 best models with the lowest Akaike information criterion (AIC) and Bayesian information criterion (BIC) values. Finally, we completed a 10-fold cross-validation with out-of-sample predictions on these selected 1000 models. We selected the best model based on out-of-sample root mean squared error. The top-five models are shown in Table 5. Our final model is model 1 in both SSA and non-SSA sections.

Finally, we used a spatiotemporal Gaussian process regression (ST-GPR) to model government spending on malaria, independently modeling sub-Saharan African countries and non-sub-Saharan African countries. The first stage of ST-GPR was a mixed-effect model with random effects on Global Burden of Disease region and country, as well as the covariates selected using the method described above. To detect and reduce the influence of outlier data points, we used the selected model to measure Cooke's distance for each data point. We excluded each data point if Cooke's distance was greater than 4/n where n is the total number of 𝐺𝑜𝑣 𝑡𝑜𝑡𝑎𝑙𝑚𝑎𝑙 data points.

**Table S5: Top government spending models based on out-of-sample root-mean square error**

| **SSA** | **Covariates** | **OOS RMSE** |
| --- | --- | --- |
| 1 | Malaria prevalence, ACT coverage, Malaria Lysenko PFPR (Highest Endemicity), Antenatal Care (4 visits) Coverage  (proportion) | 0.751537 |
| 2 | Malaria prevalence, ACT coverage, Antenatal Care (4 visits)  Coverage (proportion) | 0.751913 |
| 3 | Malaria prevalence, ACT coverage, Malaria Lysenko PFPR (Highest Endemicity), Antenatal Care (4 visits) Coverage  (proportion), Healthcare access and quality index | 0.752229 |
| 4 | Malaria prevalence ACT coverage , Malaria Lysenko PFPR (Highest Endemicity), Antenatal Care (4 visits) Coverage (proportion) , Skilled Birth Attendance (proportion) | 0.752298 |
| 5 | Malaria prevalence , ACT coverage , Antenatal Care (4 visits) Coverage (proportion) , Healthcare access and quality index | 0.752587 |
| **Non - SSA** | **Covariates** | **OOS RMSE** |
| 1 | PFPR adjusted for ITN and IRS coverage, Malaria Lysenko PFPR (Epidemic), urbanicity, Skilled Birth Attendance (proportion) | 0.824391 |
| 2 | PFPR adjusted for ITN and IRS coverage, Malaria Lysenko PFPR (Epidemic), Skilled Birth Attendance (proportion) | 0.824412 |
| 3 | PFPR adjusted for ITN and IRS coverage, urbanicity, Skilled Birth Attendance (proportion) | 0.824696 |

|  |  |  |
| --- | --- | --- |
| 4 | PFPR adjusted for ITN and IRS coverage, Skilled Birth Attendance (proportion) | 0.824799 |
| 5 | Malaria Lysenko PFPR (Epidemic), urbanicity, Skilled Birth Attendance (proportion) | 0.824862 |

## Modeling disaggregated government spending on malaria

To estimate a full time series for all years and countries in our study, we modelled each disaggregated malaria category as a share of total government spending on health with ST-GPR. We first considered the following covariates: malaria incidence, malaria prevalence, LDI per capita, coverage of indoor residual spraying (IRS), insecticide-treated nets (ITNs), and ACTs, the plasmodium falciparum parasite rate (PFPR), PFPR adjusted for IRS and ITNS, malaria Lysenko 1, 2 and 5 measures, one- and four-visit antenatal care coverage, the healthcare access and quality index (HAQI), the proportion of the population living in an urban area, coverage of skilled birth attendance (SBA), the sociodemographic index (SDI) and the universal health coverage (UHC) index. All the covariates measured as

a proportion were logit-transformed. We sourced all covariate estimates from the GBD Study 2021 and from Malaria Atlas Project.

Because the “Other” category was consistently and comparatively a lower proportion in sub-Saharan Africa, the lack of information available regarding the “Other” category, and patterns of incidence and intervention strategies differ from other regions, we split the government malaria spending data on other into two groups – sub-Saharan African and non-sub-Saharan African countries – and modeled each group separately. The remaining disaggregated malaria spending categories were modeled with data from all regions. We performed covariate selection for each of the 12 datasets, starting with the set of 19 potential covariates.

We first conducted a lasso regression to determine which covariates were least correlated, conditional on other covariates, with the fraction of government spending on malaria as the dependent variable. Covariates with an estimated coefficient of zero were removed from the set of possible covariates. We then used linear mixed effects regression to estimate all models including all possible combinations of the remaining covariates.

We then selected the intersection of 1000 best models with the lowest Akaike information criterion (AIC) and Bayesian information criterion (BIC) values. Finally, we completed a 10-fold cross-validation with out-of-sample predictions on these selected 1000 models. We selected the best model based on out-of-sample root mean squared error for each dataset. The top model for each disaggregated malaria spending category are shown in Table S6.

Finally, we used a spatiotemporal Gaussian process regression (ST-GPR) to model government spending on malaria, independently modeling each disaggregated malaria spending category. The first stage of ST-GPR was a mixed-effect model with random effects on Global Burden of Disease region and country, as well as the covariates selected using the method described above. To detect and reduce the influence of outlier data points, we used the selected model to measure Cooke's distance for each data point. We excluded each data point if Cooke's distance was greater than 4/n where n is the total number of data points for each individual dataset.

**Table S6: Top disaggregated government spending models based on out-of-sample root-mean square error**

| **Disaggregated malaria spending category** | **Covariates** |
| --- | --- |
| Anti-malarial medicines | IRS coverage (proportion), antenatal care (1+ visits) coverage (proportion), urbanicity, socio-demographic index, universal health coverage, malaria prevalence |
| Communication and advocacy | IRS coverage (proportion), ACT coverage (proportion), antenatal care (4+ visits) coverage (proportion), malaria incidence |
| Diagnostics | Malaria prevalence |
| Human resources and technical assistance + Training | ITN coverage (proportion), ACT coverage (proportion), antenatal care (4+ visits) coverage (proportion), urbanicity, socio-demographic index, malaria incidence, lag distributed income per capita |
| Infrastructure and equipment | ACT coverage (proportion), antenatal care (1+ visits) coverage (proportion), healthcare access and quality index, socio-demographic index, malaria incidence, lag distributed income per capita |
| Insecticide and spraying materials | IRS coverage (proportion), ACT coverage (proportion), antenatal care (4+ visits) coverage (proportion), universal health coverage, malaria prevalence, lag distributed income per capita |
| ITNs/PBOs | ITN coverage (proportion), malaria prevalence, lag distributed income per capita |
| Monitoring and evaluation | Urbanicity, malaria incidence, lag distributed income per capita |
| Planning, administration, overheads | Antenatal care (1+ visits) coverage (proportion), skilled birth attendance (proportion), universal health coverage, malaria incidence |
| Procurement and supply management | ACT coverage (proportion), healthcare access and quality index, socio-demographic index, malaria incidence, malaria prevalence, lag distributed income per capita |
| Other – sub-Saharan Africa | ITN coverage (proportion), antenatal care (1+ visits) coverage (proportion), malaria incidence, lag distributed income per capita |
| Other – non-sub-Saharan Africa | ITN coverage (proportion), lag distributed income per capita |

# S4: Spending efficiency analysis

## Data

## Malaria outcome estimates

We leveraged multiple sets of estimates to perform the spending efficiency analysis. We leveraged country-year-specific estimates of incident malaria cases and deaths attributed to malaria as the two primary outcome variables in the frontier analysis portion of the spending efficiency analysis. These data are preliminary estimates from the Global Burden of Disease Study 2021 (GBD). Between the 2019 and 2021 iterations of the study, methodology has largely remained consistent. For the 2021 iteration, a COVID-19 adjustment was employed to account for the impact of the pandemic. The incident case estimates have been adjusted for the COVID-19 pandemic. The mortality estimates have not been adjusted for the COVID-19 pandemic as this data was not available at the time of analysis. This adjustment was derived from the PULSE surveys employed by the WHO. The adjustment was applied to antimalarial effective treatment rates, which are inputs in modelling malaria morbidity. This adjustment was employed for the following 33 African countries included in our analysis: Angola, Burundi, Benin, Burkina Faso, Central African Republic, Côte d'Ivoire, Cameroon, Democratic Republic of the Congo, Congo, Ethiopia, Gabon, Ghana, Guinea, Guinea-Bissau, Equatorial Guinea, Kenya, Liberia, Madagascar, Mali, Mozambique, Malawi, Niger, Nigeria, Rwanda, Sudan, Sierra Leone, Somalia, South Sudan, Chad, Togo, United Republic of Tanzania, Uganda, and Zambia. For comparability between countries, we calculated a cumulative incidence rate by dividing the number of incident malaria cases by the country-year-specific population at risk of malaria infection, and we calculated a case fatality ratio by dividing the number of deaths attributed to malaria by the country-year-specific number of incident cases. Estimates of population at risk were sourced from the Malaria Atlas Project (MAP).

$${malaria incidence rate}=\frac{{\# of incident malaria cases}_{GBD}}{{population at risk}_{MAP}}$$

$${malaria case fatality ratio}=\frac{{\# of deaths attributed to malaria}_{GBD}}{{population at risk}_{MAP}}$$

To create values that best reflect the impact of 10-year spending estimates, we calculated two 10-year change in outcome values for each country of interest. We subtracted the 2020 values by the 2010 values and the 2010 values by the 2000 values for both malaria incidence rate and case fatality ratios. We log-transformed the results, which produced two values per country of interest as dependent variables in our analysis. Countries with no incident malaria cases in 2010 and 2020 were excluded from the analysis.

## Total health expenditure on malaria estimates

We leveraged country-year-specific estimates of total health expenditure on malaria from the initial portion of this study as the independent variable in the frontier analysis portion of the spending efficiency analysis. For comparability between countries, we divided total spending on malaria by the country-year-specific population at risk of malaria infection. To create ten-year spending estimates, we calculated the average malaria spending per person at risk for 2001 to 2010 and 2011 to 2020. We determined that spending between 2011 and 2020 impacted 2020 outcomes, and spending between 2001 and 2010 impacted 2010 outcomes. Although we produced spending estimates for 2000, these values were excluded from the analysis to remain consistent in the number of years included in the independent variable. We log-transformed these values. These values were our predictor variable in creating the frontiers as the first portion of our spending efficiency analysis.

## Covariates

## Estimates leveraged for covariates were extracted from the same sources as described in S3.4 of the supplementary appendix. The same estimates were included as potential covariates for the spending efficiency analysis. Additionally, population-weighted annual rainfall was included as a potential covariate in the spending efficiency analysis. Population-weighted annual rainfall was sourced from the Global Burden of Disease Study 2021. Covariate estimates were averaged over the time periods 2001 to 2010 and 2011 to 2020 to align with the spending estimates used as the independent variable in the frontier analysis.

## Government health expenditure on malaria, development assistance for malaria, and disaggregated health expenditure on malaria estimates

## We leveraged country-year-specific estimates of government health expenditure on malaria, development assistance for malaria, and disaggregated health expenditure on malaria from the initial portion of this study as predictor variables in the regression portion of the spending efficiency analysis. We calculated the sum of each of these country-specific estimates over the time periods 2001 to 2010 and 2011 to 2020 to align with the spending estimates used as the independent variable in the frontier analysis. The sum of government health expenditure on malaria and development assistance for malaria were divided by the sum of total health expenditure on malaria over the same time periods to create proportions of spending on malaria by financing source. These values were leveraged in the first regression analysis focusing on the impact of financing sources on spending efficiency. The sum of the disaggregated health expenditure on malaria estimates by spending type were divided by the sum of the government health expenditure on malaria and development assistance for malaria to create proportions of spending on malaria by spending type. These values were leveraged in the second regression analysis focusing on the impact of spending types on spending efficiency.

## Frontier analysis

## Frontier analysis is an economic modeling method typically used to examine efficiency in the production process. It models the optimal output level for a product or service given an input level. In the context of the frontier analysis, the distance between a combination of input/output point and the frontier is defined as the measure of “inefficiency”. In Figure S1, the gap between firm A’s input/output value and the frontier represents a measure of the additional output that could be produced if firm A’s production process was more efficiently organized like firm B, whose production point is on the frontier and produces a higher output level with the same input level as firm A.

## Figure S1: Intuition on frontier analysis

##
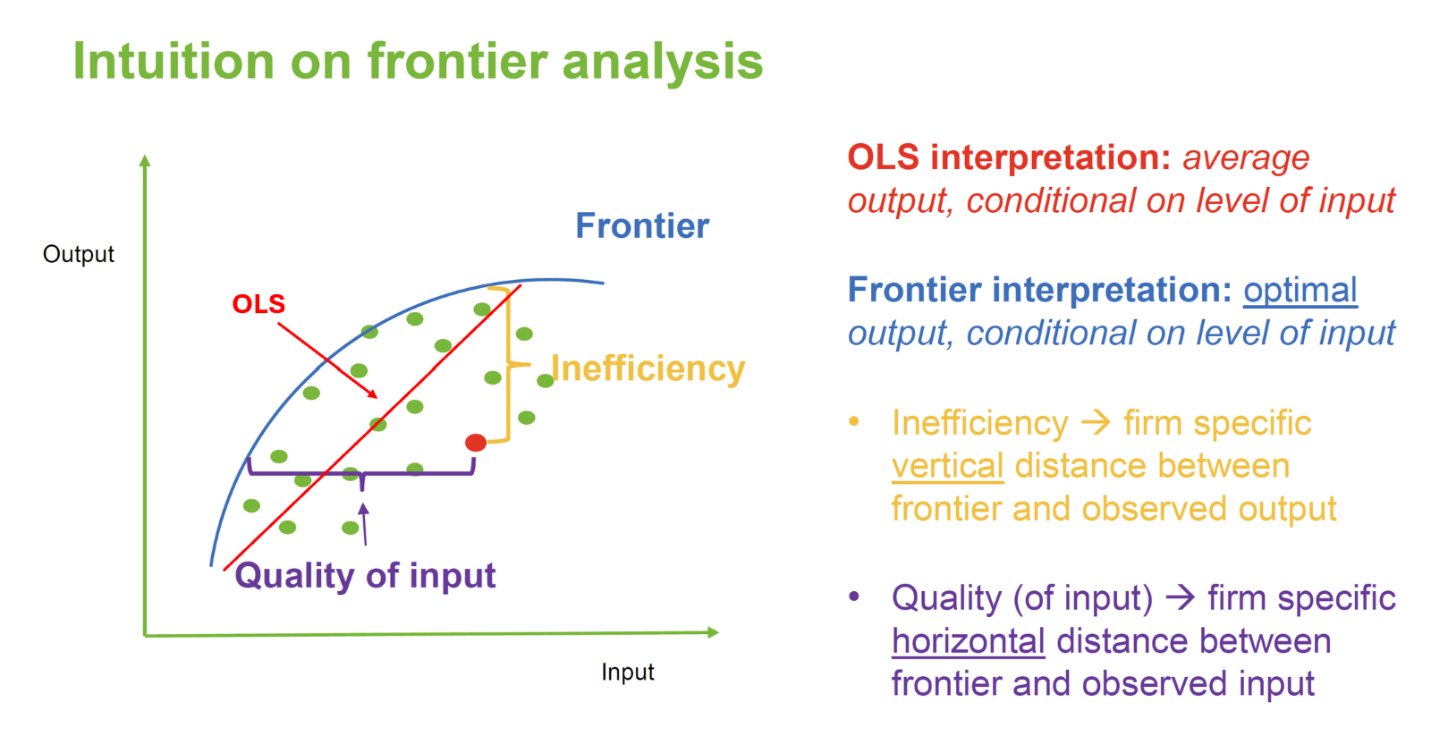


## The methodology for the creation of the frontiers is explained elsewhere.^70^ 10-year average total health expenditure per person at risk of malaria infection was the independent variable. 10-year change in malaria incidence rate or case fatality ratio were the dependent variable for the two frontier analyses. We explored the inclusion of the covariates from the estimate creation portion of this study. In addition, we considered population-weighted rainfall and regional incidence rates exclusive of the country for which the covariate would be applied. We calculated the average of these values over the time periods 2001 to 2010 and 2011 to 2020 to align with the spending variable (independent variable). We completed Bayesian Model Selection, which leveraged every potential combination of covariates to assess the associations with each covariate. Covariates that had odds of inclusion greater than 0.8 were further considered. We checked the directionality of beta for these covariates and removed those that did meet expectations set apriori. Ultimately, rainfall was selected as a covariate in the incidence-spending frontier and no covariates were selected for the case fatality-spending frontier.

## Country exemplar analysis

## Estimates of inefficiency calculated as the vertical distance from the covariate-adjusted spending-outcome estimate to the frontier were leveraged in the country exemplar analysis (Table S7). Trimmed estimates output from creating the frontier were excluded from the analysis. The average inefficiency values were calculated for each country. The countries with the lowest average inefficiency values for each frontier analysis (10-year change in incidence and 10-year change in case fatality ratio) were selected as country exemplars in incidence-spending efficiency and case fatality-spending efficiency. They were ranked in ascending order of inefficiency.

## Table S7: All country exemplar analysis estimates and frontier line estimates

## Incidence

| Country | 10 year period | Average health expenditure on malaria per person at risk (logarithmic space) | Inefficiency estimate | Covariate adjusted 10-year long difference in malaria outcome measure (logarithmic space) | Frontier estimate | Estimate type |
| --- | --- | --- | --- | --- | --- | --- |
| China | 2010 | -3.94 | 1.32 | -7.4 | -8.77 | Estimate |
| Democratic People's Republic of Korea | 2010 | -1.51 | 2.26 | -7.93 | -10.2 | Estimate |
| Democratic People's Republic of Korea | 2020 | -1.36 | 3.35 | -6.92 | -10.29 | Estimate |
| Cambodia | 2010 | 0.22 | 4 | -7.2 | -11.21 | Estimate |
| Cambodia | 2020 | 0.53 | 5.08 | -6.31 | -11.39 | Estimate |
| Indonesia | 2010 | -1.94 | 4.95 | -4.99 | -9.94 | Estimate |
| Indonesia | 2020 | -1.44 | 3.17 | -7.06 | -10.24 | Estimate |
| Lao People's Democratic Republic | 2010 | 1.21 | 4.38 | -7.4 | -11.79 | Estimate |
| Lao People's Democratic Republic | 2020 | 0.89 | 4.06 | -7.53 | -11.6 | Estimate |
| Malaysia | 2010 | 2.95 | 5.23 | -7.58 | -12.81 | Estimate |
| Myanmar | 2010 | -1.49 | 4.99 | -5.18 | -10.21 | Estimate |
| Myanmar | 2020 | -0.12 | 2.97 | -8.03 | -11.02 | Estimate |
| Philippines | 2010 | -1.95 | 3.58 | -6.35 | -9.94 | Estimate |
| Philippines | 2020 | -1.54 | 3.28 | -6.89 | -10.18 | Estimate |
| Sri Lanka | 2010 | 0.18 | 0 | -11.62 | -11.19 | Trimmed estimate |
| Thailand | 2010 | -0.46 | 4.11 | -6.7 | -10.81 | Estimate |
| Thailand | 2020 | 0.41 | 3.31 | -8.01 | -11.32 | Estimate |
| Timor-Leste | 2010 | 1.33 | 7.5 | -4.35 | -11.87 | Estimate |
| Timor-Leste | 2020 | 1.64 | 0 | -15.75 | -12.05 | Trimmed estimate |
| Viet Nam | 2010 | -1.43 | 2.69 | -7.54 | -10.25 | Estimate |
| Viet Nam | 2020 | -1.62 | 1.88 | -8.25 | -10.13 | Estimate |
| Papua New Guinea | 2010 | -0.64 | 4.48 | -6.22 | -10.71 | Estimate |
| Papua New Guinea | 2020 | 0.3 | 6.03 | -5.22 | -11.26 | Estimate |
| Solomon Islands | 2010 | 1.95 | 6.23 | -5.66 | -12.23 | Estimate |
| Solomon Islands | 2020 | 1.72 | 6.63 | -5.45 | -12.09 | Estimate |
| Vanuatu | 2010 | 2.04 | 5.61 | -6.63 | -12.28 | Estimate |
| Vanuatu | 2020 | 1.05 | 2.67 | -9.02 | -11.7 | Estimate |
| Azerbaijan | 2010 | 2.07 | 2.14 | -10.14 | -12.3 | Estimate |
| Georgia | 2010 | 0.94 | 0 | -15.17 | -11.63 | Trimmed estimate |
| Kyrgyzstan | 2010 | -2.78 | 2.14 | -7.29 | -9.45 | Estimate |
| Tajikistan | 2010 | -0.98 | 0 | -12.67 | -10.51 | Trimmed estimate |
| Turkmenistan | 2010 | 0.54 | 1.24 | -10.15 | -11.4 | Estimate |
| Uzbekistan | 2010 | 3.22 | 0 | -14.51 | -12.97 | Trimmed estimate |
| Republic of Korea | 2010 | -1.16 | 2.54 | -7.85 | -10.4 | Estimate |
| Republic of Korea | 2020 | -1.03 | 3.41 | -7.06 | -10.48 | Estimate |
| Argentina | 2010 | 2.76 | 0.12 | -12.59 | -12.7 | Estimate |
| Belize | 2010 | 0.08 | 2.74 | -8.37 | -11.13 | Estimate |
| Dominican Republic | 2010 | -1.1 | 5.52 | -4.88 | -10.44 | Estimate |
| Dominican Republic | 2020 | -0.86 | 3.56 | -7 | -10.58 | Estimate |
| Guyana | 2010 | 0.84 | 5.44 | -6.11 | -11.57 | Estimate |
| Guyana | 2020 | 0.84 | 6.41 | -5.15 | -11.58 | Estimate |
| Haiti | 2010 | -0.45 | 5.9 | -4.83 | -10.82 | Estimate |
| Haiti | 2020 | -0.72 | 4.25 | -6.35 | -10.66 | Estimate |
| Suriname | 2010 | 3.07 | 4.7 | -8.15 | -12.88 | Estimate |
| Suriname | 2020 | 3.05 | 5.2 | -7.62 | -12.88 | Estimate |
| Bolivia (Plurinational State of) | 2010 | 0.03 | 4.48 | -6.59 | -11.1 | Estimate |
| Bolivia (Plurinational State of) | 2020 | -0.09 | 5.45 | -5.51 | -11.03 | Estimate |
| Ecuador | 2010 | 2.19 | 2.57 | -9.78 | -12.37 | Estimate |
| Ecuador | 2020 | 2.26 | 6.96 | -5.41 | -12.41 | Estimate |
| Peru | 2010 | -0.21 | 4.64 | -6.3 | -10.96 | Estimate |
| Peru | 2020 | -0.63 | 5.28 | -5.41 | -10.71 | Estimate |
| Colombia | 2010 | 0.75 | 5.08 | -6.43 | -11.52 | Estimate |
| Colombia | 2020 | 0.41 | 5.45 | -5.86 | -11.32 | Estimate |
| Costa Rica | 2010 | 1.6 | 2.79 | -9.21 | -12.02 | Estimate |
| Costa Rica | 2020 | 1.35 | 5.28 | -6.57 | -11.88 | Estimate |
| El Salvador | 2010 | 0.84 | 1.86 | -9.7 | -11.58 | Estimate |
| Guatemala | 2010 | -0.28 | 2.48 | -8.42 | -10.92 | Estimate |
| Guatemala | 2020 | -0.65 | 3.15 | -7.53 | -10.7 | Estimate |
| Honduras | 2010 | -1.16 | 2.87 | -7.52 | -10.4 | Estimate |
| Honduras | 2020 | -2.09 | 1.94 | -7.91 | -9.85 | Estimate |
| Mexico | 2010 | 2.53 | 4.94 | -7.6 | -12.57 | Estimate |
| Mexico | 2020 | 2.6 | 5.99 | -6.59 | -12.61 | Estimate |
| Nicaragua | 2010 | 0.28 | 1.67 | -9.57 | -11.24 | Estimate |
| Nicaragua | 2020 | 0.62 | 9.61 | -1.81 | -11.45 | Estimate |
| Panama | 2010 | 0.35 | 4.1 | -7.16 | -11.29 | Estimate |
| Panama | 2020 | 0.38 | 6.97 | -4.3 | -11.31 | Estimate |
| Venezuela (Bolivarian Republic of) | 2010 | -0.72 | 5.38 | -5.25 | -10.66 | Estimate |
| Venezuela (Bolivarian Republic of) | 2020 | -1.45 | 6.55 | -3.65 | -10.23 | Estimate |
| Brazil | 2010 | 1 | 5.17 | -6.49 | -11.67 | Estimate |
| Brazil | 2020 | 0.47 | 5.46 | -5.88 | -11.36 | Estimate |
| Paraguay | 2010 | 2.87 | 1.05 | -11.7 | -12.77 | Estimate |
| Algeria | 2010 | 1.3 | 4.63 | -6.93 | -11.85 | Estimate |
| Iran (Islamic Republic of) | 2010 | 3.55 | 5.26 | -7.89 | -13.16 | Estimate |
| Iran (Islamic Republic of) | 2020 | 3.08 | 5.61 | -7.28 | -12.89 | Estimate |
| Iraq | 2010 | 0.6 | 0 | -11.44 | -11.44 | Trimmed estimate |
| Oman | 2010 | -0.49 | 6.26 | -4.54 | -10.8 | Estimate |
| Oman | 2020 | -0.9 | 5.77 | -3.06 | -10.55 | Estimate |
| Saudi Arabia | 2010 | 3.09 | 3.35 | -9.54 | -12.9 | Estimate |
| Saudi Arabia | 2020 | 2.77 | 10.2 | -2.51 | -12.71 | Estimate |
| Türkiye | 2010 | 1.15 | 0 | -12.6 | -11.76 | Trimmed estimate |
| Yemen | 2010 | -0.93 | 4.7 | -5.74 | -10.54 | Estimate |
| Yemen | 2020 | -1.36 | 6.43 | -3.79 | -10.28 | Estimate |
| Afghanistan | 2010 | -1.84 | 4.24 | -5.73 | -10 | Estimate |
| Afghanistan | 2020 | -0.91 | 5.29 | -5.23 | -10.55 | Estimate |
| Bangladesh | 2010 | -1.38 | 3.53 | -6.71 | -10.27 | Estimate |
| Bangladesh | 2020 | -0.35 | 3.06 | -7.8 | -10.88 | Estimate |
| Bhutan | 2010 | 0.37 | 2.45 | -8.83 | -11.3 | Estimate |
| Bhutan | 2020 | 0.5 | 2.4 | -8.97 | -11.38 | Estimate |
| India | 2010 | -2.05 | 4.21 | -5.67 | -9.88 | Estimate |
| India | 2020 | -2.16 | 2.42 | -7.4 | -9.82 | Estimate |
| Nepal | 2010 | -1.36 | 3.48 | -6.77 | -10.28 | Estimate |
| Nepal | 2020 | -1.43 | 0.72 | -9.52 | -10.24 | Estimate |
| Pakistan | 2010 | -2.82 | 4.33 | -5.08 | -9.43 | Estimate |
| Pakistan | 2020 | -2.11 | 4.59 | -5.23 | -9.84 | Estimate |
| Angola | 2010 | 0.3 | 5.32 | -5.9 | -11.26 | Estimate |
| Angola | 2020 | 0.68 | 6.4 | -5.03 | -11.48 | Estimate |
| Central African Republic | 2010 | -0.04 | 5.5 | -5.54 | -11.06 | Estimate |
| Central African Republic | 2020 | 0.92 | 5.72 | -5.82 | -11.62 | Estimate |
| Congo | 2010 | 0.62 | 5.27 | -6.13 | -11.45 | Estimate |
| Congo | 2020 | 1.8 | 6.54 | -5.5 | -12.14 | Estimate |
| Democratic Republic of the Congo | 2010 | 0.2 | 5.55 | -5.65 | -11.2 | Estimate |
| Democratic Republic of the Congo | 2020 | 0.91 | 5.69 | -5.92 | -11.62 | Estimate |
| Equatorial Guinea | 2010 | 2.62 | 6.72 | -5.87 | -12.62 | Estimate |
| Equatorial Guinea | 2020 | 3.22 | 6.66 | -6.18 | -12.97 | Estimate |
| Gabon | 2010 | 2.18 | 6.4 | -5.76 | -12.37 | Estimate |
| Gabon | 2020 | 2.33 | 5.89 | -6.33 | -12.45 | Estimate |
| Burundi | 2010 | 0.77 | 5.2 | -6.29 | -11.53 | Estimate |
| Burundi | 2020 | 1.06 | 6.68 | -4.96 | -11.7 | Estimate |
| Comoros | 2010 | 1.22 | 5.45 | -3.88 | -11.8 | Estimate |
| Comoros | 2020 | 1.15 | 3.25 | -8.48 | -11.76 | Estimate |
| Djibouti | 2010 | 0.94 | 6.84 | -4.75 | -11.63 | Estimate |
| Djibouti | 2020 | 1.59 | 10.97 | -1.01 | -12.02 | Estimate |
| Eritrea | 2010 | 0.25 | 5.66 | -5.3 | -11.23 | Estimate |
| Eritrea | 2020 | 0.98 | 6.27 | -5.37 | -11.66 | Estimate |
| Ethiopia | 2010 | 0.12 | 6.27 | -4.75 | -11.15 | Estimate |
| Ethiopia | 2020 | 0.33 | 4.5 | -6.76 | -11.28 | Estimate |
| Kenya | 2010 | 0.89 | 5.01 | -6.6 | -11.61 | Estimate |
| Kenya | 2020 | 0.95 | 6.06 | -5.58 | -11.64 | Estimate |
| Madagascar | 2010 | -0.65 | 4.21 | -6.48 | -10.7 | Estimate |
| Madagascar | 2020 | -0.1 | 5.41 | -5.55 | -11.02 | Estimate |
| Malawi | 2010 | 0.89 | 6.11 | -5.47 | -11.61 | Estimate |
| Malawi | 2020 | 1.57 | 5.98 | -5.97 | -12 | Estimate |
| Mozambique | 2010 | 0.01 | 5.49 | -5.6 | -11.09 | Estimate |
| Mozambique | 2020 | 1.11 | 6.11 | -5.61 | -11.74 | Estimate |
| Rwanda | 2010 | 0.6 | 4.82 | -6.47 | -11.43 | Estimate |
| Rwanda | 2020 | 1.45 | 5.85 | -6.08 | -11.94 | Estimate |
| Somalia | 2010 | -2.58 | 2.91 | -6.63 | -9.57 | Estimate |
| Somalia | 2020 | -2.46 | 5.21 | -4.4 | -9.64 | Estimate |
| United Republic of Tanzania | 2010 | 0.73 | 5.27 | -6.23 | -11.51 | Estimate |
| United Republic of Tanzania | 2020 | 1.47 | 6.34 | -5.57 | -11.94 | Estimate |
| Uganda | 2010 | 1.14 | 6.1 | -5.64 | -11.75 | Estimate |
| Uganda | 2020 | 1.48 | 5.77 | -6.17 | -11.95 | Estimate |
| Zambia | 2010 | 0.61 | 5.32 | -6.11 | -11.44 | Estimate |
| Zambia | 2020 | 1.78 | 6.73 | -5.35 | -12.13 | Estimate |
| Botswana | 2010 | -0.47 | 4.48 | -6.29 | -10.8 | Estimate |
| Botswana | 2020 | 0.7 | 5.88 | -5.59 | -11.49 | Estimate |
| Namibia | 2010 | 1.57 | 2.66 | -8.83 | -12.01 | Estimate |
| Namibia | 2020 | 1.95 | 9.17 | -3.02 | -12.23 | Estimate |
| South Africa | 2010 | 1.11 | 7.29 | -4.42 | -11.74 | Estimate |
| South Africa | 2020 | 1.44 | 6.6 | -5.3 | -11.93 | Estimate |
| Eswatini | 2010 | 1.54 | 4.34 | -7.61 | -11.99 | Estimate |
| Eswatini | 2020 | 2.33 | 6.71 | -5.7 | -12.45 | Estimate |
| Zimbabwe | 2010 | 0.87 | 5.4 | -6.02 | -11.59 | Estimate |
| Zimbabwe | 2020 | 1.39 | 6.78 | -5.04 | -11.9 | Estimate |
| Benin | 2010 | 0.64 | 5.99 | -5.43 | -11.46 | Estimate |
| Benin | 2020 | 1.26 | 6.4 | -5.4 | -11.82 | Estimate |
| Burkina Faso | 2010 | 0.73 | 6.19 | -5.32 | -11.51 | Estimate |
| Burkina Faso | 2020 | 1.64 | 6.31 | -5.69 | -12.04 | Estimate |
| Cameroon | 2010 | 1.32 | 6.09 | -5.75 | -11.86 | Estimate |
| Cameroon | 2020 | 1.63 | 6.27 | -5.73 | -12.04 | Estimate |
| Cabo Verde | 2010 | 0.89 | 5.33 | -6.25 | -11.61 | Estimate |
| Chad | 2010 | -0.63 | 5.34 | -5.3 | -10.71 | Estimate |
| Chad | 2020 | 0.63 | 6.02 | -5.27 | -11.45 | Estimate |
| Côte d'Ivoire | 2010 | 1.29 | 6.21 | -5.62 | -11.84 | Estimate |
| Côte d'Ivoire | 2020 | 1.72 | 5.88 | -6.13 | -12.09 | Estimate |
| Gambia | 2010 | 0.74 | 5.95 | -5.4 | -11.52 | Estimate |
| Gambia | 2020 | 1.47 | 5.54 | -6.38 | -11.94 | Estimate |
| Ghana | 2010 | 1.25 | 6.28 | -5.53 | -11.82 | Estimate |
| Ghana | 2020 | 2.18 | 5.98 | -6.36 | -12.36 | Estimate |
| Guinea | 2010 | 0.11 | 5.42 | -5.71 | -11.15 | Estimate |
| Guinea | 2020 | 0.91 | 5.71 | -5.85 | -11.62 | Estimate |
| Guinea-Bissau | 2010 | 0.31 | 4.38 | -6.83 | -11.27 | Estimate |
| Guinea-Bissau | 2020 | 1.4 | 5.55 | -6.06 | -11.9 | Estimate |
| Liberia | 2010 | 1.9 | 6.11 | -6.06 | -12.2 | Estimate |
| Liberia | 2020 | 2.78 | 6.88 | -5.77 | -12.72 | Estimate |
| Mali | 2010 | 1.36 | 6.41 | -5.46 | -11.88 | Estimate |
| Mali | 2020 | 1.33 | 6.3 | -5.52 | -11.86 | Estimate |
| Mauritania | 2010 | -0.41 | 4.92 | -5.66 | -10.84 | Estimate |
| Mauritania | 2020 | 0.79 | 6.92 | -4.59 | -11.55 | Estimate |
| Niger | 2010 | 0.16 | 6.17 | -4.89 | -11.18 | Estimate |
| Niger | 2020 | 0.84 | 6.16 | -5.3 | -11.57 | Estimate |
| Nigeria | 2010 | 1.02 | 6.12 | -5.55 | -11.68 | Estimate |
| Nigeria | 2020 | 1.58 | 6.31 | -5.68 | -12.01 | Estimate |
| São Tomé and Príncipe | 2010 | 1.57 | 3.72 | -8.27 | -12.01 | Estimate |
| São Tomé and Príncipe | 2020 | 2.59 | 5.77 | -6.82 | -12.61 | Estimate |
| Senegal | 2010 | 0.44 | 5.38 | -5.89 | -11.34 | Estimate |
| Senegal | 2020 | 1.07 | 5.44 | -6.26 | -11.71 | Estimate |
| Sierra Leone | 2010 | 0.24 | 5.5 | -5.7 | -11.23 | Estimate |
| Sierra Leone | 2020 | 0.93 | 5.57 | -6 | -11.63 | Estimate |
| Togo | 2010 | 0.54 | 5.57 | -5.81 | -11.4 | Estimate |
| Togo | 2020 | 1.36 | 6.12 | -5.7 | -11.88 | Estimate |
| South Sudan | 2010 | 0.02 | 5.06 | -5.99 | -11.09 | Estimate |
| South Sudan | 2020 | 0.24 | 5.71 | -5.41 | -11.22 | Estimate |
| Sudan | 2010 | 0.33 | 5.26 | -5.98 | -11.28 | Estimate |
| Sudan | 2020 | 0.63 | 7.06 | -4.24 | -11.46 | Estimate |

## Case fatality

| Country | 10 year period | Average health expenditure on malaria per person at risk (logarithmic space) | Inefficiency estimate | Covariate adjusted 10-year long difference in malaria outcome measure (logarithmic space) | Frontier estimate | Estimate type |
| --- | --- | --- | --- | --- | --- | --- |
| China | 2010 | -3.94 | 2.14 | 1.5 | -1.3 | Estimate |
| Democratic People's Republic of Korea | 2010 | -1.51 | 4.54 | 2.03 | -2.54 | Estimate |
| Democratic People's Republic of Korea | 2020 | -1.36 | 3.31 | 0.73 | -2.62 | Estimate |
| Cambodia | 2010 | 0.22 | 3.74 | 0.39 | -3.42 | Estimate |
| Cambodia | 2020 | 0.53 | 0.8 | -2.72 | -3.58 | Estimate |
| Indonesia | 2010 | -1.94 | 2.63 | 0.37 | -2.32 | Estimate |
| Indonesia | 2020 | -1.44 | 1.45 | -0.36 | -2.58 | Estimate |
| Lao People's Democratic Republic | 2010 | 1.21 | 4.92 | 1.07 | -3.92 | Estimate |
| Lao People's Democratic Republic | 2020 | 0.89 | 1.96 | -1.66 | -3.76 | Estimate |
| Malaysia | 2010 | 2.95 | 4.84 | 0.14 | -4.81 | Estimate |
| Myanmar | 2010 | -1.49 | 2.99 | 0.53 | -2.55 | Estimate |
| Myanmar | 2020 | -0.12 | 2.02 | -1.08 | -3.25 | Estimate |
| Philippines | 2010 | -1.95 | 2.23 | 0.54 | -2.32 | Estimate |
| Philippines | 2020 | -1.54 | 0.12 | -2.25 | -2.52 | Estimate |
| Sri Lanka | 2010 | 0.18 | 0.82 | -2.54 | -3.4 | Estimate |
| Thailand | 2010 | -0.46 | 3.35 | 0.32 | -3.07 | Estimate |
| Thailand | 2020 | 0.41 | 0.79 | -2.67 | -3.52 | Estimate |
| Timor-Leste | 2010 | 1.33 | 4.88 | 0.97 | -3.99 | Estimate |
| Timor-Leste | 2020 | 1.64 | 1.34 | -1.93 | -4.14 | Estimate |
| Viet Nam | 2010 | -1.43 | 2.94 | 0.42 | -2.58 | Estimate |
| Viet Nam | 2020 | -1.62 | 1.07 | -1.35 | -2.48 | Estimate |
| Papua New Guinea | 2010 | -0.64 | 3.63 | 0.68 | -2.98 | Estimate |
| Papua New Guinea | 2020 | 0.3 | 2.65 | -0.58 | -3.46 | Estimate |
| Solomon Islands | 2010 | 1.95 | 4.41 | 0.46 | -4.3 | Estimate |
| Solomon Islands | 2020 | 1.72 | 2.52 | -1.54 | -4.18 | Estimate |
| Vanuatu | 2010 | 2.04 | 4.95 | 0.68 | -4.35 | Estimate |
| Vanuatu | 2020 | 1.05 | 1.44 | -2.34 | -3.84 | Estimate |
| Azerbaijan | 2010 | 2.07 | 8.85 | 4.6 | -4.36 | Estimate |
| Georgia | 2010 | 0.94 | 0 | -4.05 | -3.79 | Trimmed estimate |
| Kyrgyzstan | 2010 | -2.78 | 0 | -7.16 | -1.89 | Trimmed estimate |
| Tajikistan | 2010 | -0.98 | 0 | -3.74 | -2.81 | Trimmed estimate |
| Uzbekistan | 2010 | 3.22 | 0 | -6.48 | -4.95 | Trimmed estimate |
| Republic of Korea | 2010 | -1.16 | 4.28 | 1.57 | -2.72 | Estimate |
| Republic of Korea | 2020 | -1.03 | 3.71 | 0.94 | -2.78 | Estimate |
| Argentina | 2010 | 2.76 | 0 | -7.01 | -4.72 | Trimmed estimate |
| Belize | 2010 | 0.08 | 2.37 | -0.89 | -3.35 | Estimate |
| Dominican Republic | 2010 | -1.1 | 1.63 | -1.08 | -2.75 | Estimate |
| Dominican Republic | 2020 | -0.86 | 3.21 | 0.57 | -2.87 | Estimate |
| Guyana | 2010 | 0.84 | 3.5 | -0.17 | -3.73 | Estimate |
| Guyana | 2020 | 0.84 | 2.6 | -0.84 | -3.74 | Estimate |
| Haiti | 2010 | -0.45 | 2.71 | -0.29 | -3.08 | Estimate |
| Haiti | 2020 | -0.72 | 2.12 | -0.62 | -2.94 | Estimate |
| Suriname | 2010 | 3.07 | 3.97 | -0.79 | -4.87 | Estimate |
| Bolivia (Plurinational State of) | 2010 | 0.03 | 3.39 | 0.15 | -3.33 | Estimate |
| Bolivia (Plurinational State of) | 2020 | -0.09 | 0.12 | -3.13 | -3.26 | Estimate |
| Ecuador | 2010 | 2.19 | 2.35 | -2.02 | -4.42 | Estimate |
| Ecuador | 2020 | 2.26 | 2.62 | -1.44 | -4.46 | Estimate |
| Peru | 2010 | -0.21 | 0.92 | -2.26 | -3.2 | Estimate |
| Peru | 2020 | -0.63 | 3.04 | 0.42 | -2.99 | Estimate |
| Colombia | 2010 | 0.75 | 2.88 | -0.74 | -3.69 | Estimate |
| Colombia | 2020 | 0.41 | 2.96 | -0.21 | -3.52 | Estimate |
| Costa Rica | 2010 | 1.6 | 6.96 | 2.89 | -4.12 | Estimate |
| Costa Rica | 2020 | 1.35 | 4.18 | 0.21 | -4 | Estimate |
| El Salvador | 2010 | 0.84 | 7.13 | 3.46 | -3.74 | Estimate |
| Guatemala | 2010 | -0.28 | 1.08 | -2.05 | -3.16 | Estimate |
| Honduras | 2010 | -1.16 | 2.9 | 0.28 | -2.72 | Estimate |
| Honduras | 2020 | -2.09 | 2.52 | 0.42 | -2.24 | Estimate |
| Mexico | 2010 | 2.53 | 5.78 | 1.21 | -4.6 | Estimate |
| Mexico | 2020 | 2.6 | 5.24 | 0.64 | -4.63 | Estimate |
| Nicaragua | 2010 | 0.28 | 4.1 | 0.75 | -3.45 | Estimate |
| Nicaragua | 2020 | 0.62 | 3.52 | 0.32 | -3.62 | Estimate |
| Panama | 2010 | 0.35 | 2.86 | -0.57 | -3.49 | Estimate |
| Venezuela (Bolivarian Republic of) | 2010 | -0.72 | 2.72 | -0.15 | -2.94 | Estimate |
| Venezuela (Bolivarian Republic of) | 2020 | -1.45 | 2.11 | -0.3 | -2.57 | Estimate |
| Brazil | 2010 | 1 | 2.97 | -0.78 | -3.82 | Estimate |
| Brazil | 2020 | 0.47 | 2.04 | -0.85 | -3.55 | Estimate |
| Paraguay | 2010 | 2.87 | 10.45 | 5.77 | -4.77 | Estimate |
| Algeria | 2010 | 1.3 | 3.42 | -0.09 | -3.97 | Estimate |
| Iran (Islamic Republic of) | 2010 | 3.55 | 4.25 | -0.58 | -5.12 | Estimate |
| Iran (Islamic Republic of) | 2020 | 3.08 | 1.74 | -2.22 | -4.88 | Estimate |
| Iraq | 2010 | 0.6 | 0 | -4.48 | -3.61 | Trimmed estimate |
| Oman | 2010 | -0.49 | 0 | -5.02 | -3.06 | Trimmed estimate |
| Oman | 2020 | -0.9 | 2.82 | 1.77 | -2.85 | Estimate |
| Saudi Arabia | 2010 | 3.09 | 4.91 | 0.18 | -4.88 | Estimate |
| Saudi Arabia | 2020 | 2.77 | 2.22 | -2.06 | -4.72 | Estimate |
| Syrian Arab Republic | 2010 | -3.18 | 0 | -6.06 | -1.69 | Trimmed estimate |
| Türkiye | 2010 | 1.15 | 0 | -4.2 | -3.89 | Trimmed estimate |
| Yemen | 2010 | -0.93 | 3.21 | 0.58 | -2.83 | Estimate |
| Yemen | 2020 | -1.36 | 1.77 | -0.58 | -2.61 | Estimate |
| Afghanistan | 2010 | -1.84 | 3.01 | 0.83 | -2.37 | Estimate |
| Afghanistan | 2020 | -0.91 | 1 | -1.73 | -2.85 | Estimate |
| Bangladesh | 2010 | -1.38 | 2.74 | 0.19 | -2.6 | Estimate |
| Bangladesh | 2020 | -0.35 | 2.37 | -0.52 | -3.13 | Estimate |
| Bhutan | 2010 | 0.37 | 3.02 | -0.41 | -3.5 | Estimate |
| India | 2010 | -2.05 | 2.12 | -0.13 | -2.26 | Estimate |
| India | 2020 | -2.16 | 2.39 | 0.23 | -2.21 | Estimate |
| Nepal | 2010 | -1.36 | 3.24 | 0.69 | -2.61 | Estimate |
| Nepal | 2020 | -1.43 | 1.44 | -0.93 | -2.58 | Estimate |
| Pakistan | 2010 | -2.82 | 2.24 | 0.43 | -1.87 | Estimate |
| Pakistan | 2020 | -2.11 | 0.94 | -1.05 | -2.23 | Estimate |
| Angola | 2010 | 0.3 | 3.47 | 0.09 | -3.46 | Estimate |
| Angola | 2020 | 0.68 | 3.03 | -0.34 | -3.66 | Estimate |
| Central African Republic | 2010 | -0.04 | 3.34 | 0.1 | -3.29 | Estimate |
| Central African Republic | 2020 | 0.92 | 3.7 | 0.25 | -3.78 | Estimate |
| Congo | 2010 | 0.62 | 3.44 | -0.12 | -3.62 | Estimate |
| Congo | 2020 | 1.8 | 3.86 | -0.06 | -4.23 | Estimate |
| Democratic Republic of the Congo | 2010 | 0.2 | 3.19 | -0.21 | -3.41 | Estimate |
| Democratic Republic of the Congo | 2020 | 0.91 | 3.52 | -0.02 | -3.77 | Estimate |
| Equatorial Guinea | 2010 | 2.62 | 4.17 | -0.43 | -4.65 | Estimate |
| Equatorial Guinea | 2020 | 3.22 | 4.58 | 0.05 | -4.95 | Estimate |
| Gabon | 2010 | 2.18 | 3.81 | -0.38 | -4.42 | Estimate |
| Gabon | 2020 | 2.33 | 4.4 | 0.56 | -4.5 | Estimate |
| Burundi | 2010 | 0.77 | 3.65 | 0 | -3.7 | Estimate |
| Burundi | 2020 | 1.06 | 3.23 | -0.3 | -3.85 | Estimate |
| Comoros | 2010 | 1.22 | 2.09 | -0.49 | -3.93 | Estimate |
| Comoros | 2020 | 1.15 | 3.08 | -0.67 | -3.89 | Estimate |
| Djibouti | 2010 | 0.94 | 4 | 0.32 | -3.79 | Estimate |
| Djibouti | 2020 | 1.59 | 1.77 | -1.99 | -4.12 | Estimate |
| Eritrea | 2010 | 0.25 | 3.2 | 0.12 | -3.44 | Estimate |
| Eritrea | 2020 | 0.98 | 3.35 | -0.26 | -3.81 | Estimate |
| Ethiopia | 2010 | 0.12 | 2.9 | -0.32 | -3.37 | Estimate |
| Ethiopia | 2020 | 0.33 | 3.13 | -0.01 | -3.48 | Estimate |
| Kenya | 2010 | 0.89 | 3.44 | -0.3 | -3.76 | Estimate |
| Kenya | 2020 | 0.95 | 4.1 | 0.5 | -3.79 | Estimate |
| Madagascar | 2010 | -0.65 | 3.68 | 0.76 | -2.98 | Estimate |
| Madagascar | 2020 | -0.1 | 2.79 | -0.19 | -3.26 | Estimate |
| Malawi | 2010 | 0.89 | 3.03 | -0.69 | -3.76 | Estimate |
| Malawi | 2020 | 1.57 | 3.59 | -0.15 | -4.11 | Estimate |
| Mozambique | 2010 | 0.01 | 3.04 | -0.25 | -3.31 | Estimate |
| Mozambique | 2020 | 1.11 | 3.18 | -0.39 | -3.88 | Estimate |
| Rwanda | 2010 | 0.6 | 3.37 | -0.04 | -3.61 | Estimate |
| Rwanda | 2020 | 1.45 | 3.47 | -0.39 | -4.05 | Estimate |
| Somalia | 2010 | -2.58 | 1.82 | -0.12 | -1.99 | Estimate |
| Somalia | 2020 | -2.46 | 2.15 | 0.27 | -2.05 | Estimate |
| United Republic of Tanzania | 2010 | 0.73 | 3.5 | -0.14 | -3.68 | Estimate |
| United Republic of Tanzania | 2020 | 1.47 | 3.24 | -0.52 | -4.06 | Estimate |
| Uganda | 2010 | 1.14 | 3.5 | -0.36 | -3.89 | Estimate |
| Uganda | 2020 | 1.48 | 3.6 | -0.13 | -4.06 | Estimate |
| Zambia | 2010 | 0.61 | 3.36 | -0.21 | -3.62 | Estimate |
| Zambia | 2020 | 1.78 | 3.59 | -0.25 | -4.22 | Estimate |
| Botswana | 2010 | -0.47 | 2.48 | -0.52 | -3.07 | Estimate |
| Botswana | 2020 | 0.7 | 2.95 | -0.46 | -3.66 | Estimate |
| Namibia | 2010 | 1.57 | 3.25 | 0.22 | -4.11 | Estimate |
| Namibia | 2020 | 1.95 | 2.54 | -1.08 | -4.3 | Estimate |
| South Africa | 2010 | 1.11 | 3.43 | -0.38 | -3.88 | Estimate |
| South Africa | 2020 | 1.44 | 3.03 | -0.37 | -4.04 | Estimate |
| Eswatini | 2010 | 1.54 | 3.89 | -0.11 | -4.09 | Estimate |
| Eswatini | 2020 | 2.33 | 2.22 | -1.64 | -4.49 | Estimate |
| Zimbabwe | 2010 | 0.87 | 4.06 | 0.54 | -3.75 | Estimate |
| Zimbabwe | 2020 | 1.39 | 2.95 | -0.83 | -4.02 | Estimate |
| Benin | 2010 | 0.64 | 3.78 | 0.22 | -3.63 | Estimate |
| Benin | 2020 | 1.26 | 3.29 | -0.44 | -3.95 | Estimate |
| Burkina Faso | 2010 | 0.73 | 3.69 | 0.03 | -3.68 | Estimate |
| Burkina Faso | 2020 | 1.64 | 3.76 | -0.16 | -4.14 | Estimate |
| Cameroon | 2010 | 1.32 | 3.98 | 0.04 | -3.98 | Estimate |
| Cameroon | 2020 | 1.63 | 3.79 | -0.08 | -4.14 | Estimate |
| Cabo Verde | 2010 | 0.89 | 3.26 | -0.43 | -3.76 | Estimate |
| Chad | 2010 | -0.63 | 2.7 | -0.19 | -2.99 | Estimate |
| Chad | 2020 | 0.63 | 3.31 | 0.04 | -3.63 | Estimate |
| Côte d'Ivoire | 2010 | 1.29 | 4.12 | 0.18 | -3.96 | Estimate |
| Côte d'Ivoire | 2020 | 1.72 | 3.9 | 0.04 | -4.18 | Estimate |
| Gambia | 2010 | 0.74 | 3.28 | -0.25 | -3.68 | Estimate |
| Gambia | 2020 | 1.47 | 3.68 | -0.25 | -4.05 | Estimate |
| Ghana | 2010 | 1.25 | 4.01 | 0.1 | -3.95 | Estimate |
| Ghana | 2020 | 2.18 | 4.3 | 0.14 | -4.42 | Estimate |
| Guinea | 2010 | 0.11 | 3.49 | 0.16 | -3.36 | Estimate |
| Guinea | 2020 | 0.91 | 3.23 | -0.27 | -3.77 | Estimate |
| Guinea-Bissau | 2010 | 0.31 | 3.71 | 0.34 | -3.47 | Estimate |
| Guinea-Bissau | 2020 | 1.4 | 3.35 | -0.05 | -4.02 | Estimate |
| Liberia | 2010 | 1.9 | 4.03 | -0.17 | -4.28 | Estimate |
| Liberia | 2020 | 2.78 | 4.22 | -0.1 | -4.73 | Estimate |
| Mali | 2010 | 1.36 | 4.08 | 0.1 | -4 | Estimate |
| Mali | 2020 | 1.33 | 3.63 | -0.13 | -3.99 | Estimate |
| Mauritania | 2010 | -0.41 | 2.85 | 0.06 | -3.1 | Estimate |
| Mauritania | 2020 | 0.79 | 3.15 | -0.25 | -3.71 | Estimate |
| Niger | 2010 | 0.16 | 3.2 | -0.06 | -3.39 | Estimate |
| Niger | 2020 | 0.84 | 3.33 | -0.09 | -3.73 | Estimate |
| Nigeria | 2010 | 1.02 | 3.86 | 0.06 | -3.83 | Estimate |
| Nigeria | 2020 | 1.58 | 3.48 | -0.22 | -4.11 | Estimate |
| São Tomé and Príncipe | 2010 | 1.57 | 4.65 | 0.62 | -4.11 | Estimate |
| São Tomé and Príncipe | 2020 | 2.59 | 3.38 | -1.09 | -4.63 | Estimate |
| Senegal | 2010 | 0.44 | 3.3 | -0.16 | -3.53 | Estimate |
| Senegal | 2020 | 1.07 | 3.23 | -0.52 | -3.86 | Estimate |
| Sierra Leone | 2010 | 0.24 | 3.1 | -0.29 | -3.43 | Estimate |
| Sierra Leone | 2020 | 0.93 | 3.28 | -0.13 | -3.78 | Estimate |
| Togo | 2010 | 0.54 | 3.85 | 0.33 | -3.58 | Estimate |
| Togo | 2020 | 1.36 | 3.71 | -0.01 | -4 | Estimate |
| South Sudan | 2010 | 0.02 | 3.4 | 0.14 | -3.32 | Estimate |
| South Sudan | 2020 | 0.24 | 3.35 | 0.24 | -3.43 | Estimate |
| Sudan | 2010 | 0.33 | 3.65 | 0.26 | -3.48 | Estimate |
| Sudan | 2020 | 0.63 | 3.01 | -0.3 | -3.63 | Estimate |

## Inefficiency regression analysis

# Estimates of inefficiency calculated as the vertical distance from the covariate-adjusted spending-outcome estimate to the frontier were leveraged in the inefficiency regression analysis. Two regression analyses were completed: (1) regression of proportions of financing sources on inefficiency, and (2) regression of proportions of spending types on inefficiency. The healthcare access and quality index sourced from the Global Burden of Disease Study 2021 was leveraged as a covariate in both regression analyses to best remove the impact of the health system and isolate the effects of spending source or type. Additionally, an indicator variable for the two time periods (2001-2010 and 2011-2020) was leveraged as a covariate to best remove the impact of time and technological advancements that may have affected incidence and case fatality.

# We leveraged eight of the eleven and seven of the eleven disaggregated spending types for the regression of proportions of spending types on incidence-spending inefficiency and case fatality-spending inefficiency, respectively. We aggregated estimates of ITNs/PBOs and insecticides and spraying materials to create a prevention spending type. Prevention, diagnostics, and a “catch-all” category for the remaining spending types were leveraged in the incidence-spending inefficiency regression. The catch-all category did not include anti-malarial medicines, human resources and technical assistance, and other from the estimate creation portion of the study. We wanted to prioritize investigating the impact of prevention spending on incidence-spending inefficiency. Anti-malarial medicines, diagnostics, and a “catch-all” category for the remaining spending types were leveraged in the case fatality-spending inefficiency regression. The catch-all category did not include prevention, human resources and technical assistance, and other from the estimate creation portion of the study. The resulting beta coefficients for each proportion were analyzed in each regression. Negative values were associated with decreases in inefficiency (increased efficiency), positive values were associated with increases in inefficiency (decreased efficiency).

# We conducted two additional sensitivity analyses to test the robustness of our regressions and to examine different relationships between spending and efficiency. The first sensitivity analysis was conducted on the subset of countries in Sub-Saharan Africa to examine potential different results within this region (Table S8). The second sensitivity analysis was conducted on the original set of countries with additional fixed effects for income (GDP per capita) and governance (government effectiveness index from the World Bank Group) (Table S9).

# **Table S8: Sensitivity analysis 1 – Sub-Saharan Africa**

# **Panel A. Financing sources, malaria incidence, and case fatality**

# **
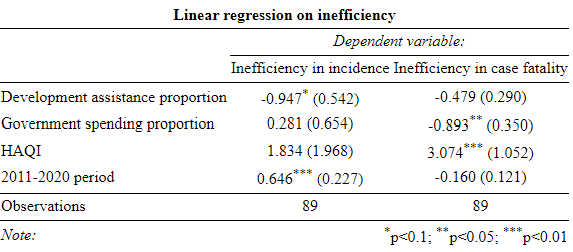
**

# **Panel B. Program areas, malaria incidence, and case fatality**

# **
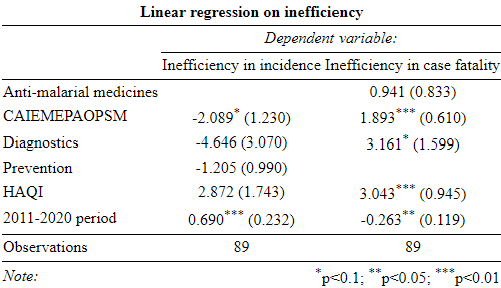
**

# **Table S9: Sensitivity analysis 2 – Income and governance**

# **Panel A. Financing sources, malaria incidence, and case fatality**

#
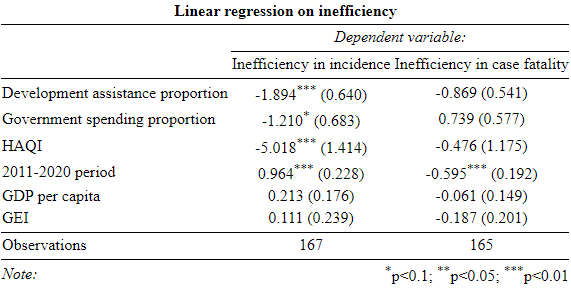


# **Panel B. Program areas, malaria incidence, and case fatality**

#
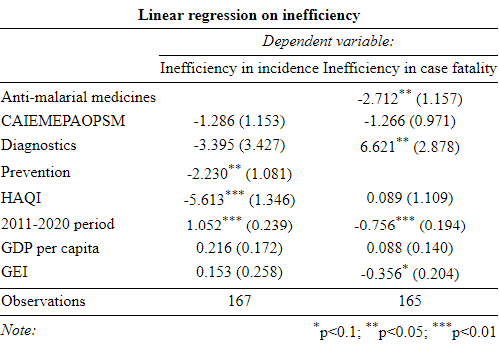


# S5: Malaria incidence categorization of the 106 countries

**Table S10: Malaria incidence categorization by country**

| **Malaria incidence in 2020** | **Countries** |
| --- | --- |
| High incidence (>30 cases per 1,000) | Angola  Benin  Burkina Faso  Burundi  Cameroon  Central African Republic  Chad  Congo  Côte d'Ivoire  Democratic Republic of the Congo  Djibouti  Equatorial Guinea  Ethiopia  Gabon  Gambia  Ghana  Guinea  Guinea-Bissau  Guyana  Kenya  Liberia  Madagascar  Malawi  Mali  Mauritania  Mozambique  Niger  Nigeria  Papua New Guinea  Rwanda  Senegal  Sierra Leone  Solomon Islands  Somalia  South Sudan  Sudan  Togo  Uganda  United Republic of Tanzania  Yemen  Zambia  Zimbabwe |
| Low incidence (<30 cases per 1,000) | Afghanistan  Bangladesh  Bhutan  Bolivia (Plurinational State of)  Botswana  Brazil  Cambodia  Colombia  Comoros  Costa Rica  Democratic People’s Republic of Korea  Dominican Republic  Ecuador  Eritrea  Eswatini  Guatemala  Haiti  Honduras  India  Indonesia  Iran (Islamic Republic of)  Lao People’s Democratic Republic  Mexico  Myanmar  Namibia  Nepal  Nicaragua  Oman  Pakistan  Panama  Peru  Philippines  Republic of Korea  São Tomé and Príncipe  Saudi Arabia  South Africa  Suriname  Thailand  Timor-Leste  Vanuatu  Venezuela (Bolivarian Republic of)  Viet Nam |
| Malaria-free (0 cases per 1,000) | Algeria  Argentina  Armenia  Azerbaijan  Belize  Cabo Verde  China  Egypt  El Salvador  Georgia  Iraq  Kazakhstan  Kyrgyzstan  Malaysia  Morocco  Paraguay  Sri Lanka  Syrian Arab Republic  Tajikistan  Turkmenistan  Türkiye  Uzbekistan |

# S6: Data Density

**Table S11: Availability of data by source and country elimination status**

| **Source** | **Control** | **Eliminating** | **Malaria-Free** | **Total** |
| --- | --- | --- | --- | --- |
| ACTwatch | 124 | 21 |  | 145 |
| Affordable Medicine Facility -  Malaria | 127 |  |  | 127 |
| Battle et al.  (2016) | 1598 | 1394 | 544 | 3536 |
| Global Fund  Concept Notes | 73 | 55 | 6 | 134 |
| Global Fund Price & Quality  Reporting | 3135 | 973 | 47 | 4155 |
| Global Fund  Proposals | 131 | 81 | 12 | 224 |
| Health Action  International | 207 | 21 | 1 | 229 |
| Moses et al.  (2018) | 1598 | 1394 | 612 | 3604 |
| NHA | 120 | 29 |  | 149 |
| WHO Global Price Reporting  Mechanism | 16074 | 2301 | 79 | 18454 |
| WMR | 2224 | 2367 | 690 | 5281 |
| Total | 25411 | 8636 | 1991 | 36038 |

**Table S12: Availability of data by source and GBD super-region**

| **Source** | **Central Europe, Eastern Europe, and Central**  **Asia** | **High- income** | **Latin America and Caribbean** | **North Africa and Middle East** | **South Asia** | **Southeast Asia, East Asia, and Oceania** | **Sub- Saharan Africa** | **Total** |
| --- | --- | --- | --- | --- | --- | --- | --- | --- |
| ACTwatch |  |  |  |  |  | 11 | 134 | 145 |
| Affordable Medicine Facility -  Malaria |  |  |  |  |  |  | 127 | 127 |
| Battle et al.  (2016) | 238 | 68 | 646 | 374 | 170 | 510 | 1530 | 3536 |

| Global Fund Concept  Notes | 2 |  | 14 | 5 | 8 | 28 | 77 | 134 |
| --- | --- | --- | --- | --- | --- | --- | --- | --- |
| Global Fund Price & Quality  Reporting | 37 |  | 66 | 166 | 240 | 517 | 3129 | 4155 |
| Global Fund  Proposals | 12 |  | 24 | 15 | 15 | 38 | 120 | 224 |
| Health Action  International |  |  | 3 | 22 | 4 | 15 | 185 | 229 |
| Moses et al.  (2018) | 272 | 68 | 646 | 408 | 170 | 510 | 1530 | 3604 |
| NHAs |  |  |  | 2 |  | 9 | 138 | 149 |
| WHO Global Price Reporting  Mechanism | 69 |  | 604 | 917 | 254 | 1784 | 14826 | 18454 |
| WMRs | 317 | 96 | 1125 | 457 | 293 | 867 | 2126 | 5281 |
| Total | 947 | 232 | 3128 | 2366 | 1154 | 4289 | 23922 | 36038 |

# S7: GATHER compliance

This study complies with the Guidelines for Accurate and Transparent Health Estimates Reporting (GATHER) recommendations.3 We have documented the steps involved in our analytical procedures and detailed the data sources used. See Table 3 for the GATHER checklist.

The GATHER recommendations can be found here: http://gather-statement.org/

| Table 9. GATHER Compliance Checklist # | GATHER checklist item | Description of compliance | Reference |
| --- | --- | --- | --- |
| Objectives and funding | | | |
| 1 | Define the indicators, populations, and time periods for which estimates were made. | Narrative provided in paper and methods appendix describing  indicators, definitions, and populations | Main text (Methods—  Overview,  Geographical units and time periods) and methods appendix |
| 2 | List the funding sources for the work. | Funding sources listed in paper | Main text (Acknowledgments) |
| Data Inputs | | | |
| *For all data inputs from multiple sources that are synthesized as part of the study:* | | | |
| 3 | Describe how the data were identified and how the data were accessed. | Narrative provided in paper and methods appendix describing data-seeking methods | Main text (Methods) and methods appendix |
| 4 | Specify the inclusion and exclusion criteria. Identify all ad-hoc exclusions. | Narrative provided in paper and methods appendix describing inclusion and exclusion criteria | Main text (Methods) and methods appendix |
| 5 | Provide information on all included data sources and their main characteristics. For each data source used, report reference information or contact name/institution, population represented, data collection method, year(s) of data collection, sex and age range, diagnostic criteria or measurement method, and sample size, as relevant. | Metadata for data sources by component, activity, geography, currency, currency year, and income classification will be available through an interactive, online data record | Link to the GHDx to be provided upon publication. |

# S8: References

1. World Development Indicators (WDI) | Data Catalog. https://datacatalog.worldbank.org/dataset/world-development-indicators (accessed Jan 31, 2019).
2. World Economic Outlook Database October 2018. https://[www.imf.org/external/pubs/ft/weo/2018/02/weodata/index.aspx](http://www.imf.org/external/pubs/ft/weo/2018/02/weodata/index.aspx) (accessed Jan 31, 2019).
3. Robert C. Feenstra RI. Penn World Table 9.0. 2016. DOI:10.15141/S5J01T.
4. National Accounts - Analysis of Main Aggregates (AMA). https://unstats.un.org/unsd/snaama/Index (accessed Jan 31, 2019).
5. WHO. Global Health Observatory (GHO) data. WHO. <http://www.who.int/gho/en/> (accessed Jan 31, 2019).
6. Global Burden of Disease Health Financing Collaborator Network. 2019. Past, present and future of global health financing: a review of country spending on health and development assistance for 195 countries, 1995-2050. *The Lancet*. Submitted Manuscript.
7. World Health Organization (WHO). World Malaria Report. WHO; Geneva, Switzerland. Available at: https://[www.who.int/malaria/publications/world_malaria_report/en/](http://www.who.int/malaria/publications/world_malaria_report/en/) (Accessed January 15, 2019).
8. WHO. Global Health Expenditure Database: Documentation Centre: National Reports. WHO; Geneva, Switzerland. Available at: <http://apps.who.int/nha/database/DocumentationCentre/Index/en> (Accessed January 15, 2019.
9. Moses MW, Pedroza P, Baral R, Bloom S, Brown J, Chapin A, Compton K, Eldrenkamp E, Fullman N, Mumford JE, Nandakumar V, Rosettie K, Sadat N, Shonka T, Flaxman A, Vos T, Murray CJL, Weaver MR. 2018. Funding and services needed to achieve universal health coverage: applications of global, regional, and national estimates of utilisation of outpatient visits and inpatient admissions from 1990 to 2016, and unit costs from 1995 to 2016**.** *The Lancet Public Health*.
10. Ayieko P, Akumu AO, Griffiths UK, English M. 2009. The economic burden of inpatient paediatric care in Kenya: household and provider costs for treatment of pneumonia, malaria and meningitis. *Cost-Effectiveness and Resoure Allocation.* 7:3.
11. Davis WA, Clarke PM, Siba PM, Karunajeewa HA, Davy C, Mueller I, Davis TME. 2011. Cost- effectiveness of artemisinin combination therapy for uncomplicated malaria in children: data from Papua New Guinea. *Bulletin of the World Health Organization*. 89(3):211-220.
12. Gogtay NJ, Kadam VS, Desai S, Kamtekar KD, Dalvi SS, Kshirsagar NA. 2003. A cost-effectiveness analysis of three antimalarial treatments for acute, uncomplicated Plasmodium falciparum malaria in Mumbai, India. *Journal of Association of Physicians of India.* 51:877-879.
13. Hongoro C, McPake B. 2003. Hospital costs of high-burden diseases: malaria and pulmonary tuberculosis in a high HIV prevalence context in Zimbabwe. *Tropical Medicine and International Health.* 8(3):242-50.
14. Kyaw SS, Drake T, Ruangveerayuth R, Chierakul W, White NJ, Newton PN, Lubell Y. 2014. Cost of treating inpatient falciparum malaria on the Thai-Myanmar border. *Malaria Journal.* 13:416.
15. Lubell Y, Riewpaiboon A, Dondorp AM, von Seidlein L, Mokuolu OA, Nansumba M, Gesase S, Kent A, Mtove G, Olaosebikan R *et al*. 2011. Cost-effectiveness of parenteral artesunate for treating children with severe malaria in sub-Saharan Africa. *Bulletin of the World Health Organization*. 89(7):504-512.
16. Maka DE, Chiabi A, Obadeyi B, Mah E, Nguefack S, Nana P, Mbacham W, Mbonda E. 2016.

Economic evaluation of artesunate and three quinine regimens in the treatment of

severe malariain children at the Ebolowa Regional Hospital-Cameroon: a cost analysis. *Malaria Journal.* 15(1):587.

1. Muheki C, McIntyre D, Barnes KI. 2004. Artemisinin-based combination therapy reduces expenditure on malaria treatment in KwaZulu Natal, South Africa. *Tropical Medicine & International Health.* 9(9):959-966.
2. Rakuomi V, Okalebo F, Ndwigah S, Mbugua L. 2017. Cost effectiveness of pre-referral antimalarial treatment in severe malaria among children in sub-Saharan Africa. *Cost- effectiveness and Resource Allocation.* 15: 14.
3. Sicuri E, Vieta A, Lindner L, Constenla D, Sauboin C. 2013. The economic costs of malaria in children in three sub-Saharan countries: Ghana, Tanzania and Kenya. *Malaria Journal.* 12:307.
4. Svihrova V, Szilagyiova M, Krkoska D, Simekova K, Hudeckova H, Avdicova M. 2009. Analysis of the direct and indirect costs of treatment of imported malaria in the Slovak Republic. *Revista de Sociedade Brasiliera de Medicina Tropical.* 42(4):377-80.
5. Tang S, Feng D, Wang R, Ghose B, Hu T, Ji L, Wu T, Fu H, Huang Y, Feng Z. 2017. Economic burden of malaria inpatients during National Malaria Elimination Programme: estimation of hospitalization cost and its inter-province variation. *Malaria Journal.* 16(1):291. d
6. Tsakala TM, Tona GL, Mesia K, Mboma JC, Vangu JM, Voso SM, Kanja GL, Kodondi KK, Mabela M, Walo R. 2005. [Evaluation of prescriptions for inpatient treatment of malaria and gastroenteritis: Bondeko and St Joseph hospitals in Kinshasa]. [Sante.](https://www.ncbi.nlm.nih.gov/pubmed/?term=tsakala%2B2005) 15(2):119-24.
7. James SL, Abate D, Abate KH, *et al.* Global, regional, and national incidence, prevalence, and years lived with disability for 354 diseases and injuries for 195 countries and territories, 1990– 2017: a systematic analysis for the Global Burden of Disease Study 2017. *The Lancet* 2018; 392: 1789–858.
8. Battle KE, Bisanzio D, Gibson HS, Bhatt S, Cameron E, Weiss DJ, Mappin B, Dalrymple U, Howes RE, Hay SI, Gething PW. 2016. Treatment-seeking rates in malaria endemic countries. *Malaria Journal.* 15(20).
9. WHO. 2008. World malaria report 2008. WHO; Geneva, Switzerland. p. 215.
10. Deressa W. 2007. Treatment-seeking behaviour for febrile illness in an area of seasonal malaria transmission in rural Ethiopia. *Malaria Journal.* 6:49.
11. The Global Burden of Disease Financing Global Health Collaborator Network. 2019. Past, present, and future of global health financing: country spending, and development assistance for health for 195 countries, 1995–2050. *The Lancet.* Submitted Manuscript.
12. Moses MW, Pedroza P, Baral R, Bloom S, Brown J, Chapin A, Compton K, Eldrenkamp E, Fullman N, Mumford JE, Nandakumar V, Rosettie K, Sadat N, Shonka T, Flaxman A, Vos T, Murray CJL, Weaver MR. 2018. Funding and services needed to achieve universal health coverage: applications of global, regional, and national estimates of utilisation of outpatient visits and inpatient admissions from 1990 to 2016, and unit costs from 1995 to 2016**.** *The Lancet Public Health*.
13. Chanda P, Masiye F, Chitah BM, Sipilanyambe N, Hawela M, Banda P, Okorosobo T. 2007. A cost-effectiveness analysis of artemether lumefantrine for treatment of uncomplicated malaria in Zambia. *Malaria Journal.* 6.
14. Davis WA, Clarke PM, Siba PM, Karunajeewa HA, Davy C, Mueller I, Davis TME. 2011. Cost- effectiveness of artemisinin combination therapy for uncomplicated malaria in children: data from Papua New Guinea. *Bulletin of the World Health Organization*. 89(3):211-220.
15. Ezenduka CC, Falleiros DR, Godman BB. 2017. Evaluating the Treatment Costs for Uncomplicated Malaria at a Public Healthcare Facility in Nigeria and the Implications. *Pharmacoeconomics.* 1(3):185-194.
16. Liu Y, Zhang YL, Gao LJ, Qian D, Yang CY, Zhou RM, Xu BL, Zhang HW. 2016. Medical Costs on Malaria in Henan Province and Analysis of the Influential Factors. *Zhongguo Ji Sheng Chong Xue Yu Ji Sheng Chong Bing Za Zhi.* (1):11-7.
17. Muheki C, McIntyre D, Barnes KI. 2004. Artemisinin-based combination therapy reduces expenditure on malaria treatment in KwaZulu Natal, South Africa. *Tropical Medicine & International Health.* 9(9):959-966.
18. Njau JD, Goodman CA, Kachur SP, Mulligan J, Munkondya JS, McHomvu N, Abdulla S, Bloland P, Mills A. 2008. The costs of introducing artemisinin-based combination therapy: evidence from district-wide implementation in rural Tanzania. *Malaria Journal.* 7.
19. Salawu AT, Fawole OI, Dairo MD. 2016. Patronage and Cost of Malaria Treatment in Private Hospitals in Ibadan North L.G.A. South Western Nigeria. *Analysis of Ibadan Post-graduate Medicine.* 14(2):81-84.
20. Sicuri E, Vieta A, Lindner L, Constenla D, Sauboin C. 2013. The economic costs of malaria in children in three sub-Saharan countries: Ghana, Tanzania and Kenya. *Malaria Journal.* 12:307.
21. Wiseman V, Kim M, Mutabingwa TK, Whitty CJM. 2006. Cost-effectiveness study of three antimalarial drug combinations in Tanzania. *Plos Medicine.* 3:1844-1850.
22. Institute for Health Metrics and Evaluation (IHME). 2018. Financing Global Health 2017:Funding Universal Health Coverage and the Unfinished HIV/AIDS Agenda. IHME; Seattle, WA. Available at: <http://www.healthdata.org/policy-report/financing-global-health-2017> (Accessed January 16, 2019).
23. Castillo-Riquelme M, McIntyre D, Barnes K. 2008. Household burden of malaria in South Africa and Mozambique: is there a catastrophic impact? *Tropical Medicine and International*

*Health.* 13:108–122.

1. Deressa W, Hailemariam D, Ali A. 2007. Economic costs of epidemic malaria to households in rural Ethiopia. *Tropical Medicine and International Health* 12(10):1148-56.
2. Gunda R, Shamu S, Chimbari MJ, Mukaratirwa S. 2017.Economic burden of malaria on rural households in Gwanda district, Zimbabwe. *African Journal of Primary Health Care and Family Medicine.* 9(1):e1-e6.
3. Hennessee I, Chinkhumba J, Briggs-Hagen M, [Bauleni A,](https://www.ncbi.nlm.nih.gov/pubmed/?term=Bauleni%20A%5BAuthor%5D&cauthor=true&cauthor_uid=28969643) Shah MP, Chalira A, Moyo D, Dodoli W, Luhanga M, Sande J, Ali D, Gutman J, Lindblade KA, Njau J, Mathanga DP. 2017. Household costs among patients hospitalized with malaria: evidence from a national survey in Malawi, 2012. *Malaria Journal.* 16(1):395. doi: 10.1186/s12936-017-2038-y.
4. Ilunga-Ilunga F, Levêque A, Laokri S, Dramaix M. 2015. Incidence of catastrophic health expenditures for households: an example of medical attention for the treatment of severe childhood malaria in Kinshasa reference hospitals, Democratic Republic of Congo. *Journal of Infection and Public Health.* 8:136–144.
5. Nabyonga Orem J, Mugisha F, Okui AP, Musango L, Kirigia JM. 2013. Health care seeking patterns and determinants of out-of-pocket expenditure for malaria for the children under-five in Uganda. *Malaria Journal.* 12:175.
6. Onwujekwe O, Uguru N, Etiaba E, Chikezie I, Uzochukwu B, [Adjagba A](https://www.ncbi.nlm.nih.gov/pubmed/?term=Adjagba%20A%5BAuthor%5D&cauthor=true&cauthor_uid=24223796). 2013. The economic burden of malaria on households and the health system in Enugu State southeast Nigeria. *PLoS One.* 8(11):e78362.
7. Sicuri E, Vieta A, Lindner L, Constenla D, Sauboin C. 2013.The economic costs of malaria in children in three sub-Saharan countries: Ghana, Tanzania and Kenya. *Malaria Journal.* 12:307.
8. Xia S, Ma JX, Wang DQ, Li SZ, Rollinson D, Zhou SS, Zhou XN. 2016. Economic cost analysis

of malaria case management at the household level during the malaria elimination phase in The People's Republic of China. *Infectious Diseases of Poverty.* 5(1):50.

1. Castellani J, Mihaylova B, Siribié M, Gansane Z, Ouerdraogo AZ, Fouque F, Sirima SB, Evers SMAA, Paulus ATG, Gomes M. 2018. Household costs and time to treatment for children with severe febrile illness in rural Burkina Faso: the role of rectal artesunate. *Malaria Journal.* 17: 380.
2. Castillo-Riquelme M, McIntyre D, Barnes K. 2008. Household burden of malaria in South Africa and Mozambique: is there a catastrophic impact? *Tropical Medicine and International Health.* 1:108-22.
3. Chuma J, Okungu V, Molyneux C. 2010. The economic costs of malaria in four Kenyan districts: do household costs differ by disease endemicity? *Malaria Journal.* 2 (9): 149.
4. Ewing VL, Lalloo DG, Phiri KS, Roca-Feltrer A, Mangham LJ, SanJoaquin MA. 2011. Seasonal and geographic differences in treatment-seeking and household cost of febrile illness among children in Malawi. *Malaria Journal.* 10:32.
5. Matovu F, Nanyiti A, Rutebemberwa E. 2014. Household health care-seeking costs: experiences from a randomized, controlled trial of community-based malaria and pneumonia treatment among under-fives in eastern Uganda. *Malaria Journal.* 13:222.
6. Morel CM, Thang ND, Xa NX, Hung le X, Thuan le K, Van Ky P, Erhart A, Mills AJ, D'Alessandro U. 2008. The economic burden of malaria on the household in south-central Vietnam. *Malaria Journal.* 7:166.
7. Mota RE, Lara AM, Kunkwenzu ED, Lalloo DG. 2009. Health seeking behavior after fever onset in a malaria-endemic area of Malawi. *American Journal of Tropical Medicine and Hygiene.* 81(6):935-43.
8. Mugisha F, Kouyate B, Gbangou A, Sauerborn R. 2002. Examining out-of-pocket expenditure on health care in Nouna, Burkina Faso: implications for health policy. *Tropical Medicine and International Health.*7(2):187-96.
9. Mustafa MH, Babiker MA. 2007. Economic cost of malaria on households during a transmission season in Khartoum State, Sudan. *Eastern Mediterranean Health Journal.* 13(6):1298-307.
10. Nabyonga Orem J, Mugisha F, Okui AP, Musango L, Kirigia JM. 2013. Health care seeking patterns and determinants of out-of-pocket expenditure for malaria for the children under-five in Uganda. *Malaria Journal.* 12:175.
11. Onwujekwe O, Uguru N, Etiaba E, Chikezie I, Uzochukwu B, Adjagba A. 2013.The economic burden of malaria on households and the health system in Enugu State southeast Nigeria. *PLoS One.* 8(11):e78362.
12. Xia S, Ma JX, Wang DQ, Li SZ, Rollinson D, Zhou SS, Zhou XN. 2016. Economic cost analysis

of malaria case management at the household level during the malaria elimination phase in The People's Republic of China. *Infectious Disease of Poverty.* 5(1):50.

1. ACTwatch. News & Publications. Available at: <http://www.actwatch.info/publications> (Accessed January 16, 2019).
2. Health Action International (HAI). Price & Availability Data: Database of medicine prices, availability, affordability and price components. HAI. Available at: <http://haiweb.org/what-we-> do/price-availability-affordability/price-availability-data/ (Accessed January 16, 2019).
3. Arnold F, Ye Y, Ren R, Yoder S, Hanson K, Goodman C, Tougher S, Mann A, Willey B. 2012. Independent Evaluation of Phase 1 of the Affordable Medicines Facility - malaria (AMFm): multi- country independent evaluation report: final report. *The Global Fund to Fight AIDS, Tuberculosis and Malaria.* Available at: https://unitaid.org/assets/Mid-term-evaluation-Affordable- medicines-for-malaria-facility-AMFm-Phase-1.pdf. (Accessed January 18, 2019).
4. Management Sciences for Health (MSH). 2018. International Medical Products Price Guide. Available at: <http://mshpriceguide.org/en/home/> (Accessed January 16, 2019).
5. WHO. Global Price Reporting Mechanism for HIV, tuberculosis and malaria. WHO; Geneva, Switzerland. Available at: https://[www.who.int/hiv/amds/gprm/en/](http://www.who.int/hiv/amds/gprm/en/) (Accessed January 16, 2019).
6. The Global Fund to Fight AIDS, Tuberculosis and Malaria. Price & Quality Reporting. The Global Fund; Geneva, Switzerland. Available at: https://[www.theglobalfund.org/en/sourcing-](http://www.theglobalfund.org/en/sourcing-) management/price-quality-reporting/ (Accessed January 16, 2019).
7. Management Sciences for Health (MSH). 2018. International Medical Products Price Guide. Available at: <http://mshpriceguide.org/en/home/> (Accessed January 16, 2019).
8. Based on an analysis of: Management Sciences for Health (MSH). 2018. International Medical Products Price Guide. Available at: <http://mshpriceguide.org/en/home/> (Accessed January 16, 2019).
9. Bennett A, Bisanzio D, Yukich JO, Mappin B, Fergus CA, Lynch M, Cibulskis RE, Bhatt S, Weiss DJ, Cameron E, Gething PW, Eisele TP. 2017. Population coverage of artemisinin-based combination treatment in children younger than 5 years with fever and Plasmodium falciparum infection in Africa, 2003–2015: a modelling study using data from national surveys. *The Lancet Global Health.* 5:e418-27.
10. Global Burden of Disease Health Financing Collaborator Network. Past, present and future of global health financing: a review of country spending on health and development assistance for 195 countries, 1995-2050. *The Lancet*; Submitted Manuscript.
11. Dieleman JL. Kaldjian AS, Sahu M, Chen C, Liu A, Chapin A, Woody Scott K, Aravkin S, Zheng P, Mokdad A, Murray CJL, Schulman K, Milstein A. Estimating health care delivery system value for each US state and testing key associations. Health Services Research. 24 May 2021. doi: 10.1111/1475-6773.13676.
